# Three-dimensional kinematics of canine hind limbs: in vivo, biplanar, high-frequency fluoroscopic analysis of four breeds during walking and trotting

**DOI:** 10.1038/s41598-018-34310-0

**Published:** 2018-11-19

**Authors:** Martin S. Fischer, Silvia V. Lehmann, Emanuel Andrada

**Affiliations:** Institut für Zoologie und Evolutionsforschung, Friedrich-Schiller-Universität Jena, Erbertstr. 1, 07743 Jena, Germany

## Abstract

The first high-precision 3D *in vivo* hindlimb kinematic data to be recorded in normal dogs of four different breeds (Beagle, French bulldog, Malinois, Whippet) using biplanar, high-frequency fluoroscopy combined with a 3D optoelectric system followed by a markerless XROMM analysis (Scientific Rotoscoping, SR or 3D-2D registration process) reveal a) 3D hindlimb kinematics to an unprecedented degree of precision and b) substantial limitations to the use of skin marker-based data. We expected hindlimb kinematics to differ in relation to body shape. But, a comparison of the four breeds sets the French bulldog aside from the others in terms of trajectories in the frontal plane (abduction/adduction) and long axis rotation of the femur. French bulldogs translate extensive femoral long axis rotation (>30°) into a strong lateral displacement and rotations about the craniocaudal (roll) and the distal-proximal (yaw) axes of the pelvis in order to compensate for a highly abducted hindlimb position from the beginning of stance. We assume that breeds which exhibit unusual kinematics, especially high femoral abduction, might be susceptible to a higher long-term loading of the cruciate ligaments.

## Introduction

Two-dimensional descriptions of segment and joint kinematics (pro- and retraction/flexion and extension) have long been available (e.g.^1–7^). Fischer and Lilje^8^ studied kinematics in 327 dogs of 32 breeds in different gaits in the sagittal plane using a marker-based system. Their book also reports on the first extensive study using fluoroscopy to describe sagittal joint angles and segment motion in seven breeds during the walk and trot and compares the results with those of previous studies. Their focus on sagittal kinematics is explained by the logical need to first understand the direction of locomotion. However, locomotion involves three- dimensional movements of limb segments, and these movements may differ between breeds according to differences in leg morphology. Little is known about the 3D kinematics of the canine pelvic limb, and next to nothing about the possible relationship between leg morphology and 3D kinematics.

Our study is the first high-precision 3D *in vivo* investigation of total hindlimb kinematics in healthy dogs of four different breeds. It uses biplanar, high-frequency fluoroscopy and a 3D optoelectric system in combination with a newly developed marker setup. To the best of our knowledge, the two approaches closest to ours to date are the studies by Headrick *et al*.^9^ and Kim *et al*.^10^, which describe the 3D motion of the pelvic limb and determine the 3D *in vivo* kinematics of the healthy canine stifle joint, respectively. Studies on quadrupedal locomotion of mammals using biplanar fluoroscopy are sparse e.g.^10–25^. Headrick’s study was marker-based at 60 Hz and involved only four cameras, while Kim *et al*. used uniplanar fluoroscopy at 30 Hz, conceding “Data were obtained using single-plane fluoroscopic imaging, which is less accurate for measuring out-of-sagittal-plane motions than biplanar systems”. Earlier 3D *in vivo* investigations into stifle kinematics used invasive bone implants to gather kinematic data in healthy dogs^26,27^. However, both of these studies focus on the abnormal motion associated with anterior cruciate ligament ACL deficiency, and the kinematic patterns detected in healthy dogs were not described in depth. Approaches which compare fluoroscopy and marker-based recordings serve to test the accuracy of the latter (e.g.^9,28,29^).

After having demonstrated long axis rotation of about 10° for the scapula and the humerus in a study into 3D forelimb kinematics and inverse dynamics in Beagles^23^, we were curious as to how differences in body form and leg posture translate to long axis rotation.

Possible reasons for ACL degeneration as a chronic phenomenon in certain dog breeds are still under debate (e.g.^11,30^). One of the purposes of our study was to experimentally show possible *in vivo* torsion of the stifle joint during the walk and trot at least in four different breeds. In this respect, we hypothesizes that breeds having more abducted legs might display larger long axis rotation or torsion of the stifle during the stance phase (assuming no slip between foot and ground). Understanding differences in 3D kinematics between different breeds might lead to a better understanding of chronic stress in canine cruciate ligaments.

The four breeds were selected on a “strength” versus “speed” (French bulldog versus Whippet) basis, and then for their “normal” qualities (Malinois) and for pragmatic reasons (Beagle). The Malinois build is generally wolf-like in terms of proportions and body shape. Beagles (or foxhounds, see^27^) are often used in veterinary studies. The idea behind the selection of French bulldogs and Whippets is based on the work of Chase *et al*.^31^ and Carrier *et al*.^32^, who analyzed QTLs (quantitative trait loci) and revealed some unexpected connections. Trade-offs are evident in Portuguese water dogs, Greyhounds and Pitbulls. Speed types and strength types differ in skull and pelvis shape and in the transverse profile of their long bones (elliptical vs. round). Resistance to fracturing is two-fold greater in the strength type, whereas bone stiffness is 60% higher in Greyhounds. Difference in ribcage shape (either slim or round in cross-section) leads to a difference in limb position – and this is the reason for the selection of this trait. The question is how the differences in leg posture in the four breeds translate to kinematics. In line with the trade-off “strength” vs “speed”, we would expect a higher degree of segmental abduction/adduction and axial rotation in the French bulldog, and a lesser degree of pro- and retraction. Less abduction/adduction and axial rotation is expected for generalist dogs such as the Malinois and the Beagle, and an even greater degree of parasagittal motion is expected in Whippets during both the walk and the trot.

## Results

### Speed walk, speed trot, duty factor

During fluoroscopic data collection the dogs’ mean speed ± SD and mean duty factor ± SD at walk (w) and trot (t) were: Malinois: (w. 1.3 ± 0.25 m/s, 0.58 ± 0.02), (t. 2.3 ± 0.32 m/s, 0.42 ± 0.03); Whippet: (w. 0.9 ± 0.1 m/s, 0.6 ± 0.03), (t. 1.7 ± 0.19 m/s, 0.42 ± 0.05); French Bulldog: (w. 0.7 ± 0.05 m/s, 0.63 ± 0.05), (t. 1.4 ± 0.21 m/s, 0.47 ± 0.03); Beagle: (w. 0.8 ± 0 m/s, 0.6 ± 0.03), (t. 1.6 ± 0.32 m/s, 0.42 ± 0.07).

During marker-based data collection the dogs’ mean speed ± SD and mean duty factor ± SD at walk (w) and trot (t) were: Malinois: (w. 1.2 ± 0 m/s, 0.64 ± 0.02), (t. 2.5 ± 0.3 m/s, 0.42 ± 0.04); Whippet: (w. 1.0 ± 0.05 m/s, 0.56 ± 0.06), (t. 1.8 ± 0.21 m/s, 0.41 ± 0.03); French Bulldog: (w. 0.8 ± 0.05 m/s, 0.56 ± 0.06), (t. 1.5 ± 0.11 m/s, 0.41 ± 0.02); Beagle: (w. 1,0 ± 0.04 m/s, 0.61 ± 0.03), (t. 2.2 ± 0.22 m/s, 0.39 ± 0.03).

### Presentation of data

In the following, presentation of the SR data pertaining to limb segment trajectories will be followed by presentation of the marker-based data. SR data will then be qualitatively compared to marker-based data, because differences are surprisingly obvious. Only marker-based data are given for the stifle joint and lower leg of Malinois and the hock joint and foot trajectory of all four breeds because these structures could not be identified with reliable accuracy using SR.

The tests of within (gait) and between (breed) subjects effects generally showed gait, breed, and breed * gait specifics to have a significant influence on the dependent variables (at least p < 0.05). Below, we indicate when this was not the case and present the results of the post-hoc tests (more information can be found in Tables 1, 2, and 3).

**Table 1:** Mean and standard deviations of segment and joint kinematics about the latero-medial axes based on Scientific Rotoscoping and results of statistical tests.

	% Stance					% Swing		
	TD	25	50	75	TO	25	50	75
***Pelvis***								
Wh_w (1)	**49.6 ± 3**	48.6 ± 1.9	**48.0 ± 2.3**	49.8 ± 2.7	**49.6 ± 2.4**	48.3 ± 2	**47. 8 ± 1.8**	48.6 ± 2
Wh_ t (1)	**46.5 ± 3.2**	44.7 ± 3.0	**43.9 ± 2.6**	44.5 ± 2.2	**46.0 ± 2.3**	45.8 ± 3.4	**44.5 ± 3**	46.2 ± 2.3
FB_w (2)	**51.7 ± 4.8**	48.4 ± 3.4	**46.7 ± 3.2**	50.6 ± 4.3	**50.1 ± 4.2**	47.1 ± 3.8	**47.4 ± 3.7**	49.2 ± 4.2
FB_t (2)	**48.3 ± 4.2**	46.1 ± 3.6	**44.5 ± 3.2**	45.4 ± 3.3	**47.6 ± 3.6**	46.3 ± 4.1	**43.6 ± 4.8**	45.8 ± 4.9
Ma_w (3)	**56.2 ± 6**	53.7 ± 6.6	**50.3 ± 6.9**	53.5 ± 6.6	**54.2 ± 6.1**	51.1 ± 6	**49.9 ± 6.7**	52.1 ± 6.5
Ma_t (3)	**47.5 ± 5.5**	46.1 ± 5.3	**45.8 ± 5.7**	46.5 ± 5.7	**46.8 ± 5.1**	46.1 ± 4.7	**45.8 ± 5.3**	46.9 ± 5.9
Be_w (4)	**38.7 ± 4.4**	34.8 ± 4.2	**31.8 ± 4.5**	35.3 ± 3.4	**37 ± 3.1**	34.2 ± 4.6	**31.8 ± 4.9**	34.6 ± 4.6
Be_t (4)	**33.3 ± 6.2**	31.5 ± 5.9	**29.6 ± 5.1**	29.6 ± 4.5	**31.5 ± 4.9**	32.6 ± 5.5	**30.5 ± 4.1**	31.4 ± 4.1
Gait Breeds Gait*Breeds	**p < 0.001**		**p < 0.001**		**p < 0.001**		**p < 0.001**	
	**p < 0.001**		**p < 0.001**		**p < 0.001**		**p < 0.001**	
	**p = 0.03**		**p = 0.012**		**n.s**.		**n.s**.	
Post-Hoc-tests	**1** ^(3** 4***)^		**1** ^(4***)^		**1** ^(4***)^		**1** ^(4***)^	
	**2** ^(4***)^		**2** ^(4***)^		**2** ^(4***)^		**2** ^(4***)^	
	**3** ^(1** 4***)^		**3** ^(4***)^		**3** ^(4***)^		**3** ^(4***)^	
	**4** ^(1*** 2*** 3***)^		**4** ^(1*** 2*** 3***)^		**4** ^(1*** 2*** 3***)^		**4** ^(1*** 2*** 3***)^	
***Hip***								
Wh_w (1)	**−5.6 ± 6.3**	−15.9 ± 4.4	**−26.5 ± 4.6**	−42.2 ± 5.4	**−47.3 ± 5.5**	−34.2 ± 7.1	**−15.8 ± 7.1**	−4.3 ± 6.1
Wh_t (1)	**−2.9 ± 4.6**	−9.7 ± 3.2	**−17.6 ± 3.1**	−29.4 ± 5.2	**−44.5 ± 6.3**	−44.9 ± 3.9	**−12.9 ± 6.8**	5.1 ± 4.7
FB_w (2)	**0.4 ± 5.2**	−9.3 ± 3.8	**−21.0 ± 5.6**	−40.2 ± 7.5	**−33.2 ± 6.5**	−13.4 ± 6.6	**3.9 ± 5.1**	8.4 ± 5.1
FB_t (2)	**2.8 ± 5.3**	−4.5 ± 3.6	**−13.7 ± 4.9**	−26.3 ± 7.0	**−38.4 ± 7.4**	−28.9 ± 5.5	**2.7 ± 5.6**	15.3 ± 7.7
Ma_w (3)	**−8.1 ± 15.3**	−16.4 ± 14.1	**−26.0 ± 12.2**	−45.4 ± 10.4	**−55.1 ± 6.9**	−37.1 ± 6.5	**−6.0 ± 9.4**	−1.9 ± 12.4
Ma_t (3)	**−2.5 ± 10.9**	−11.3 ± 10.5	**−25 ± 11.7**	−43.3 ± 10.6	**−49.7 ± 6.5**	−32.2 ± 7.9	**−4.9 ± 8.1**	6.8 ± 10.7
Be_w (4)	**13.5 ± 5.3**	4.0 ± 6.7	**−8.7 ± 8.6**	−26.5 ± 7.6	**−36.5 ± 6.8**	−22.9 ± 9.7	**1.6 ± 8**	13.8 ± 7.1
Be_t (4)	**7.1 ± 2.8**	0.3 ± 2.7	**−8.8 ± 3.2**	−20.6 ± 3.9	**−32.3 ± 3.1**	−28.7 ± 9.6	**3.7 ± 8.6**	15.9 ± 2.8
Gait Breeds Gait*Breeds	**n.s**.		**p < 0.001**		**n.s**.		**n.s**.	
	**p < 0.001**		**p < 0.001**		**p < 0.001**		**p < 0.001**	
	**p < 0.001**		**p = 0.004**		**p < 0.001**		**p = 0.001**	
Post-Hoc-tests	**1** ^(2*** 4***)^		**1** ^(2*** 4***)^		**1** ^(2*** 3** 4***)^		**1** ^(2*** 3** 4***)^	
	**2** ^(1*** 4***)^		**2** ^(1*** 3* 4***)^		**2** ^(1*** 3***)^		**2** ^(1*** 3**)^	
	**3** ^(4***)^		**3** ^(2* 4***)^		**3** ^(1** 2*** 4***)^		**3** ^(1*** 2*** 4***)^	
	**4** ^(1*** 2*** 3***)^		**4** ^(1*** 2*** 3***)^		**4** ^(1*** 3***)^		**4** ^(1*** 3***)^	
***Stifle***								
Wh_w (1)	**−51.8 ± 3.8**	−54.9 ± 6.1	**−57.9 ± 6.9**	−54.8 ± 7.7	**−55.7 ± 9.5**	−71.4 ± 10.3	**−77.1 ± 6.7**	−66.4 ± 5.4
Wh_t (1)	**−50.9 ± 5.7**	−56.7 ± 5.2	**−62.2 ± 6.3**	−61.4 ± 6.9	**−54.0 ± 8.2**	−61.0 ± 7.0	**−92.1 ± 6.6**	−77.0 ± 5.8
FB_w (2)	**−52 ± 6.3**	−57.7 ± 7.9	**−59.6 ± 6.8**	−52 ± 5.1	**−68.6 ± 8.4**	−86.9 ± 7.3	**−86.3 ± 9.3**	−68.6 ± 9.7
FB_ t (2)	**−55.7 ± 8.2**	−61.5 ± 8.4	**−64.9 ± 7.4**	−60.5 ± 6.3	**−54.1 ± 5.6**	−77.9 ± 10.5	**−108.8 ± 9.2**	−85.4 ± 10.7
Be_w (4)	**−40.7 ± 4.5**	−45.1 ± 3	**−45.5 ± 4.9**	−42.8 ± 5.9	**−44.9 ± 7.6**	−59.5 ± 7.6	**−69.7 ± 5.1**	−58.5 ± 6.3
Be_t (4)	**−41.7 ± 5.1**	−48.0 ± 3.7	**−49.9 ± 3.8**	−46.2 ± 6	**−43.7 ± 4.6**	−67.7 ± 14.4	**−96 ± 8.6**	−69.3 ± 10.4
Gait Breeds Gait*Breeds	**p = 0.006**		**p < 0.001**		**p < 0.001**		**p < 0.001**	
	**p < 0.001**		**p < 0.001**		**p < 0.001**		**p < 0.001**	
	**n.s**.		**n.s**.		**p < 0.001**		**p = 0.043**	
Post-Hoc-tests	**1** ^(4***)^		**1** ^(4***)^		**1** ^(2*** 4***)^		**1** ^(2***)^	
	**2** ^(4***)^		**2** ^(4***)^		**2** ^(1*** 4***)^		**2** ^(1*** 4***)^	
	**----**		**----**		**----**		**----**	
	**4** ^(1*** 2***)^		**4** ^(1*** 2***)^		**4** ^(1*** 2***)^		**4** ^(2***)^	
***Femur***								
Wh_w (1)	44 ± 3.7	32.6 ± 3.9	21.4 ± 4.8	7.7 ± 5.3	2.4 ± 5	14.1 ± 6.4	32 ± 6.3	44.3 ± 4.9
Wh_t (1)	43.7 ± 4.1	35.1 ± 3.5	26.2 ± 4.5	15.1 ± 6.7	1.5 ± 8.1	1.0 ± 4.4	31.6 ± 5.2	51.3 ± 4.1
FB_w (2)	52.1 ± 3.3	39.1 ± 3.6	25.7 ± 5	10.4 ± 7	15.3 ± 6.9	33.7 ± 7	51.3 ± 4.3	57.6 ± 2.1
FB_t (2)	51.1 ± 4.2	41.6 ± 3.4	30.8 ± 4.5	19.1 ± 5.2	9.2 ± 4.9	17.3 ± 4.5	46.3 ± 7.3	61.1 ± 8.1
Ma_w (3)	48.1 ± 10.3	37.3 ± 9.3	24.4 ± 7.8	8.1 ± 6.2	−0.9 ± 5.1	14.0 ± 3.9	37.9 ± 5	50.2 ± 7.1
Ma_t (3)	45.1 ± 6.8	34.9 ± 7	20.8 ± 8.1	3.2 ± 7.3	−2.9 ± 5.5	13.9 ± 8.2	40.9 ± 6.1	53.7 ± 5.9
Be_w (4)	51.8 ± 4.2	38.5 ± 6.7	22.7 ± 7.6	8.5 ± 5.9	0.4 ± 5.3	11.2 ± 5.6	31.8 ± 9.9	47.8 ± 5.9
Be_t (4)	40.4 ± 6.1	31.9 ± 5.9	20.9 ± 6	9 ± 6.1	−0.8 ± 4	3.8 ± 4.5	33.3 ± 5.1	47.3 ± 4
***Tibia***								
Wh_w (1)	−7.5 ± 3.6	−21.4 ± 3.7	−35.3 ± 4	−46.3 ± 5.2	−55.3 ± 5.7	−57.9 ± 4.4	−45.5 ± 4	−22.0 ± 4.9
Wh_t (1)	−7.5 ± 4.4	−21.3 ± 4.8	−34.8 ± 5.7	−44.5 ± 5.1	−50.5 ± 3.5	−59.6 ± 4.4	−62.0 ± 2.8	−27.4 ± 4.2
FB_w (2)	−1.4 ± 6.6	−17.9 ± 9.1	−33.8 ± 10.3	−45.4 ± 9.8	−57.4 ± 7	−55.9 ± 6.9	−36.9 ± 10.7	−12.5 ± 8.6
FB_t (2)	−4.8 ± 5.5	−19.7 ± 7	−33.4 ± 7.5	−42.7 ± 7.8	−50.8 ± 7.1	−63.8 ± 5.8	−60.8 ± 4.5	−22.6 ± 7.8
Be_w (4)	5.2 ± 4.2	−10.1 ± 4.4	−24.7 ± 3.2	−38.5 ± 2.8	−50 ± 5.6	−54.6 ± 5.3	−44.8 ± 6.8	−17.7 ± 10.7
Be_t (4)	−2.7 ± 4.4	−17.2 ± 4.4	−29.7 ± 3.6	−37.5 ± 3	−44.7 ± 3.4	−64.1 ± 9.1	−63.9 ± 3.8	−22.9 ± 8.5

Mean and standard deviations of segment and joint kinematics are presented in degrees [°]. Bold areas display timepoints at which statistical tests were applied. SD: standard deviation, TD: touch-down, TO: toe-off, Wh: Whippet, FB: French Bulldog, Ma: Malinois, Be: Beagle, w- walk, t: trot, *p < 0.05, **p < 0.01, ***p < 0.001, n.s.: non-significant.

**Table 2:** Mean and standard deviations of segment and joint kinematics about the craniocaudal axes based on Scientific Rotoscoping and results of statistical tests.

	% Stance					% Swing		
	TD	25	50	75	TO	25	50	75
***Pelvis***								
Wh_w (1)	**−1,5 ± 3,9**	−2,4 ± 5,4	**−3,1 ± 5,6**	−3,0 ± 4,0	**−2,9 ± 3,8**	−2,8 ± 4,5	**−2,6 ± 4,2**	−2,5 ± 4,1
Wh_ t (1)	**−4,7 ± 3**	−4,6 ± 3,4	**−3,9 ± 4,2**	−3,6 ± 4,3	**−4,5 ± 3,1**	−4,9 ± 3,3	**−5,9 ± 3,5**	−6 ± 3,2
FB_w (2)	**4,5 ± 5,0**	−2,9 ± 3,8	**−0,3 ± 2.3**	5,7 ± 2,6	**12,4 ± 3,1**	11,6 ± 3,9	**8,7 ± 3,9**	6 ± 4,0
FB_t (2)	**2,3 ± 3,2**	−2,7 ± 3,7	**−5,7 ± 4,2**	−3,8 ± 3,3	**3,2 ± 2,4**	11,4 ± 2,5	**14,8 ± 2,3**	11,4 ± 3,3
Ma_w (3)	**5,9 ± 2,3**	2,2 ± 2,6	**−0,1 ± 2,8**	1,1 ± 3,0	**4,8 ± 2,6**	7,6 ± 2,3	**8,3 ± 2,5**	7,8 ± 2,9
Ma_t (3)	**2,2 ± 3,4**	−0,6 ± 3,1	**−1,8 ± 3,4**	0,4 ± 4,3	**2,4 ± 4,3**	4,3 ± 3,7	**5,6 ± 3,4**	4,2 ± 3
Be_w (4)	**6,3 ± 3,1**	1,4 ± 3,2	**0 ± 3,8**	0 ± 4,1	**0,6 ± 4,6**	3 ± 4,6	**4,3 ± 4,0**	5 ± 3,7
Be_t (4)	**−0,4 ± 3,4**	−2,3 ± 3	**−3,6 ± 2,1**	−3,1 ± 1,5	**−1,7 ± 1,5**	1 ± 1,4	**3,7 ± 1,8**	2,8 ± 3,2
Gait Species Gait*Species	**p < 0.001**		**p < 0.001**		**p < 0.001**		**n.s**.	
	**p < 0.001**		**p = 0.005**		**p < 0.001**		**p < 0.001**	
	**p = 0.016**		**p = 0.002**		**p < 0.001**		**p < 0.001**	
Post-Hoc-tests	**1** ^(2*** 3*** 4***)^		**1** ^(3*)^		**1** ^(2*** 3*** 4***)^		**1** ^(2*** 3*** 4***)^	
	**2** ^(1***)^		**2** ^(3*)^		**2** ^(1*** 3* 4***)^		**2** ^(1*** 3*** 4***)^	
	**3** ^(1***)^		**3** ^(1* 2*)^		**3** ^(1*** 2* 4***)^		**3** ^(1*** 2*** 4***)^	
	**4** ^(1***)^		**4** ^**(n.s.)**^		**4** ^(1*** 2*** 3***)^		**4** ^(1*** 2*** 3***)^	
***Hip***								
Wh_w (1)	**−9,9 ± 8**,	−7,2 ± 8,9	**−7,2 ± 9,9**	−10,4 ± 11,5	**−13,6 ± 12,5**	−11,6 ± 10,9	**−9,9 ± 9,4**	−10,1 ± 8,7
Wh_t (1)	**−6,9 ± 7,1**	−6,1 ± 7,2	**−6,0 ± 7**	−6,9 ± 7,3	**−8,7 ± 10,2**	−12,8 ± 13,1	**−11,8 ± 9,3**	−9,2 ± 7,9
FB_w (2)	**−20,4 ± 7,7**	−18,7 ± 7,5	**−25,2 ± 7,4**	−38,7 ± 10,1	**−40,6 ± 7,5**	−33,8 ± 6,8	**−27,3 ± 6,7**	−22,9 ± 6,5
FB_t (2)	**−15 ± 8,2**	−17,9 ± 8,9	**−25,5 ± 9,1**	−35,4 ± 7,3	**−42,5 ± 7,2**	−37,3 ± 9,7	**−27,6 ± 10,3**	−20,7 ± 9
Ma_w (3)	**−11,3 ± 2,5**	−10,9 ± 2,7	**−12,0 ± 3,0**	−19,3 ± 5,6	**−23,6 ± 7,1**	−16,9 ± 4,7	**−13,1 ± 3,2**	−12,3 ± 3,8
Ma_t (3)	**−8,3 ± 4,6**	−9,5 ± 5,8	**−11,2 ± 6,8**	−13,3 ± 8	**−13,6 ± 8**	−11,7 ± 5,4	**−9,8 ± 3,2**	−8,1 ± 4
Be_w (4)	**−16,4 ± 4,0**	−13,2 ± 3,2	**−11,1 ± 3,5**	−8,7 ± 3,9	**−10,4 ± 4,1**	−12,7 ± 3,6	**−14,1 ± 3,5**	−16,1 ± 4,7
Be_t (4)	**−11,4 ± 5,1**	−11,0 ± 4,1	**−12,5 ± 3,1**	−16,3 ± 3	**−18,3 ± 3,6**	−14,5 ± 3,7	**−14,7 ± 2,9**	−14,9 ± 4,7
Gait Species Gait*Species	**p < 0.001**		**p = 0.013**		**p = 0.007**		**n.s**.	
	**p < 0.001**		**p < 0.001**		**p < 0.001**		**p < 0.001**	
	**n.s**.		**n.s**.		**p < 0.001**		**p = 0.001**	
Post-Hoc-tests	**1** ^(2***)^		**1** ^(2*** 4*)^		**1** ^(2***)^		**1** ^(2***)^	
	**2** ^(1*** 3*** 2*)^		**2** ^(1*** 3*** 4***)^		**2** ^(1*** 3*** 4***)^		**2** ^(1*** 3*** 4***)^	
	**3** ^(2***)^		**3** ^(2***)^		**3** ^(2***)^		**3** ^(2***)^	
	**4** ^(2*)^		**4** ^(2***)^		**4** ^(2***)^		**4** ^(2***)^	
***Stifle***								
Wh_w (1)	**16,4 ± 5,4**	17,5 ± 6,1	**18,0 ± 6,5**	19 ± 5,7	**18,9 ± 4,9**	18,2 ± 4,7	**17,6 ± 4,9**	17,4 ± 4,8
Wh_t (1)	**14,5 ± 6,4**	16,1 ± 6,6	**18,3 ± 6,4**	19,2 ± 6	**17,2 ± 4,9**	15,7 ± 5,7	**16,8 ± 8,4**	17,5 ± 6,9
FB_w (2)	**19,7 ± 5**	20,4 ± 6	**22,2 ± 6.1**	22,4 ± 4.8	**27,5 ± 2.8**	31,9 ± 3.5	**31,5 ± 4.2**	26,1 ± 4.6
FB_ t (2)	**19,6 ± 6,5**	22,4 ± 4,6	**27,9 ± 4,4**	29 ± 5,3	**23,6 ± 3,7**	25,6 ± 5,3	**31,4 ± 4,2**	28,3 ± 4,6
Be_w (4)	**13,9 ± 4.5**	15,4 ± 6.3	**17,5 ± 5.8**	17,6 ± 6	**15,3 ± 5.4**	14,1 ± 5.6	**14,2 ± 6.2**	15,2 ± 4.6
Be_t (4)	**15,4 ± 4,2**	18,3 ± 2,8	**20,7 ± 3,2**	22,2 ± 3,6	**21,1 ± 3,1**	16,9 ± 3,6	**20,5 ± 6**	18 ± 7,6
Gait Species Gait*Species	**n.s**.		**p < 0.001**		**n.s**.		**p = 0.036**	
	**p < 0.001**		**p < 0.001**		**p < 0.001**		**p < 0.001**	
	**n.s**		**p = 0.005**		**p < 0.001**		**p = 0.014**	
Post-Hoc-tests	**1** ^(2***)^		**1** ^(2***)^		**1** ^(2***)^		**1** ^(2***)^	
	**2** ^(1*** 4***)^		**2** ^(1*** 4***)^		**2** ^(1*** 4***)^		**2** ^(1*** 4***)^	
	**----**		**----**		**----**		**----**	
	**4** ^(2***)^		**4** ^(2***)^		**4** ^(2***)^		**4** ^(2***)^	
***Femur***								
Wh_w (1)	−13,5 ± 6.8	−11,8 ± 5.6	−12,5 ± 5,2	−15,6 ± 6,9	−18,7 ± 10,8	−16,6 ± 10,2	−14,7 ± 7,3	−14,8 ± 6,4
Wh_t (1)	−13,1 ± 4,4	−12,3 ± 3,6	−11,4 ± 3,9	−12,1 ± 4,6	−14,7 ± 8,2	−19,3 ± 13,6	−19,3 ± 10,9	−16,8 ± 8
FB_w (2)	−20,2 ± 3,3	−25,9 ± 3,8	−29,8 ± 4,2	−37,3 ± 7,7	−32,5 ± 6,2	−26,5 ± 5,2	−22,9 ± 4,3	−21,2 ± 2,9
FB_t (2)	−16,9 ± 5,9	−24,9 ± 7,6	−35,3 ± 8,1	−43,4 ± 5,8	−43,4 ± 5,5	−30,1 ± 5,1	−16,9 ± 5,8	−13,4 ± 5,6
Ma_w (3)	−7,0 ± 4,8	−10,3 ± 4,3	−13,7 ± 4,3	−19,7 ± 4,7	−20,3 ± 7,1	−10,9 ± 6,3	−6,4 ± 5,8	−6,1 ± 6,2
Ma_t (3)	−8,1 ± 4,5	−12,1 ± 6,1	−15,0 ± 7	−14,9 ± 8,6	−13,1 ± 8,5	−9,4 ± 6,3	−6,2 ± 4,6	−5,9 ± 5,5
Be_w (4)	−10,7 ± 3,4	−12,6 ± 3	−11,9 ± 5	−9,6 ± 6,5	−10,8 ± 6,5	−10,7 ± 5,5	−10,7 ± 4,4	−11,8 ± 3,2
Be_t (4)	−12,0 ± 2,1	−13,5 ± 1,8	−16,3 ± 2,2	−19,6 ± 3	−20,1 ± 3,5	−13,7 ± 3,9	−11,2 ± 2,3	−12,2 ± 2,2
***Tibia***								
Wh_w (1)	3,1 ± 4,8	5,9 ± 6,2	6,7 ± 7,6	6,8 ± 8,4	5,6 ± 9,6	5,5 ± 9,9	5,9 ± 8,2	5,0 ± 5,6
Wh_t (1)	5,1 ± 3,8	6,8 ± 4,5	8,6 ± 5,2	9,3 ± 5,9	8,4 ± 6,7	5,5 ± 9,3	7,1 ± 10,9	8,7 ± 5,8
FB_w (2)	5,3 ± 4,3	5,6 ± 3,3	3,9 ± 4,1	1,0 ± 4,8	8,4 ± 3,9	13,9 ± 4,3	13,5 ± 4,8	8,9 ± 4,3
FB_t (2)	9,1 ± 4,2	10,1 ± 3,1	9,7 ± 4,3	4,7 ± 5,5	−0,9 ± 4,7	6,5 ± 6,3	21,3 ± 6,6	18,0 ± 4,6
Be_w (4)	0,5 ± 2,5	4,2 ± 2,6	7,6 ± 2,6	7,9 ± 6,9	9,0 ± 15,6	10,2 ± 14,8	7,5 ± 6,6	3,3 ± 2,7
Be_t (4)	4,0 ± 3,8	6,7 ± 2,7	7,5 ± 2,6	6,8 ± 5,2	5,9 ± 5,8	6,6 ± 3,6	11,5 ± 3,1	8,0 ± 3,6

Mean and standard deviations of segment and joint kinematics are presented in degrees [°]. Bold areas display timepoints at which statistical tests were applied. SD: standard deviation, TD: touch-down, TO: toe-off, Wh: Whippet, FB: French Bulldog, Ma: Malinois, Be: Beagle, w- walk, t: trot, *p < 0.05, **p < 0.01, ***p < 0.001, n.s.: non-significant.

**Table 3:** Mean and standard deviations of segment and joint kinematics about the distal-proximal axes based on Scientific Rotoscoping and results of statistical tests.

	% Stance					% Swing		
	TD	25	50	75	TO	25	50	75
***Pelvis***								
Wh_w (1)	**−2.6 ± 4.8**	−3.1 ± 3.7	**−2.8 ± 3.3**	−2.2 ± 4.5	**−2.3 ± 4.9**	−2.6 ± 3.6	**−3.1 ± 2.7**	−3.2 ± 3.8
Wh_ t (1)	**−0.1 ± 4.8**	−0.5 ± 4	**−0.6 ± 2.9**	−0.8 ± 2.7	**−1.2 ± 3.6**	−1.1 ± 5.2	**−0.4 ± 4.5**	−0.4 ± 4
FB_w (2)	**−5.4 ± 6**	1 ± 4.7	**8.4 ± 4.6**	10.1 ± 6.1	**3.1 ± 7.4**	−1.5 ± 7.9	**−5.4 ± 7.3**	−7.2 ± 6.4
FB_t (2)	**−3.8 ± 4.4**	−4.2 ± 5.1	**−2.4 ± 5.2**	2.6 ± 4.3	**9.1 ± 3.6**	12 ± 2.9	**8.6 ± 3.5**	2.5 ± 5.1
Ma_w (3)	**0.6 ± 2.7**	0.9 ± 2.8	**2.1 ± 3.3**	3.3 ± 3.8	**3.4 ± 3.4**	2.5 ± 2.6	**1.4 ± 3**	1.1 ± 3.3
Ma_t (3)	**−0.6 ± 5.1**	−0.1 ± 4	**0.9 ± 2.5**	1.9 ± 2	**2.1 ± 2.7**	1.8 ± 2.5	**1 ± 2**	−0.1 ± 3.6
Be_w (4)	**−2.8 ± 3.7**	−0.1 ± 3.8	**3.6 ± 4.1**	4.8 ± 3.9	**2.1 ± 3.8**	−0.7 ± 3.5	**−2.7 ± 3.4**	−3.6 ± 3
Be_t (4)	**0 ± 1.5**	0.6 ± 1.3	**2 ± 1.2**	3.8 ± 1.5	**5.1 ± 1.4**	5.2 ± 1.6	**3.2 ± 1.9**	1 ± 1.6
Gait Breeds Gait*Breeds	**n.s**.		**p = 0.011**		**p = 0.003**		**p < 0.001**	
	**p = 0.001**		**p < 0.001**		**p < 0.001**		**p = 0.003**	
	**p < 0.001**		**p < 0.001**		**p = 0.01**		**p < 0.001**	
Post-Hoc-tests	**1** ^(2**)^		**1** ^(2*** 3*** 4***)^		**1** ^(2*** 3*** 4***)^		**1** ^(2**)^	
	**2** ^(1** 4*)^		**2** ^(1***)^		**2** ^(1*** 3*** 4***)^		**2** ^(1**)^	
	**3** ^**(n.s.)**^		**3** ^(1***)^		**3** ^(1*** 2***)^		**3** ^**(n.s.)**^	
	**4** ^(2*)^		**4** ^(1***)^		**4** ^(1*** 2***)^		**4** ^**(n.s.)**^	
***Hip***								
Wh_w (1)	**7.3 ± 3.9**	6.2 ± 4.4	**5.6 ± 5.3**	4.4 ± 6.3	**4.2 ± 8.1**	2.1 ± 6.5	**2.8 ± 4.6**	5.5 ± 3.8
Wh_t (1)	**7.5 ± 4**	6.8 ± 4	**6.3 ± 4.5**	5.2 ± 4.8	**3.9 ± 7.4**	4.9 ± 9.3	**3.6 ± 2.7**	5.7 ± 3.4
FB_w (2)	**14.2 ± 6**	17.1 ± 6.3	**17 ± 6.3**	24.2 ± 7.7	**24.2 ± 6.3**	17.3 ± 6.5	**13.6 ± 7.3**	13.3 ± 6.8
FB_t (2)	**12.2 ± 6.7**	15 ± 5.9	**18.6 ± 5.5**	23.9 ± 5.4	**28.4 ± 6.5**	18.1 ± 7.4	**6.9 ± 8.6**	8.6 ± 9.3
Ma_w (3)	**7.9 ± 8.8**	8.5 ± 8.5	**10.4 ± 8.2**	14.6 ± 8.2	**16.4 ± 9.8**	9.6 ± 10.1	**6.6 ± 8.7**	7.3 ± 9.2
Ma_t (3)	**2.2 ± 9**	1.3 ± 8	**0.4 ± 8.5**	−0.8 ± 9	**−0.8 ± 8.6**	−1.1 ± 9.3	**0.5 ± 9.4**	3.5 ± 10.2
Be_w (4)	**−7.5 ± 7.8**	−6.4 ± 5.3	**−9.5 ± 4.6**	−16.2 ± 7.4	**−15.9 ± 8.7**	−11.8 ± 7.5	**−10 ± 5.1**	−9 ± 4.4
Be_t (4)	**3.6 ± 5.3**	3.5 ± 5.4	**2.3 ± 6.4**	1.3 ± 8.2	**0.2 ± 9.2**	−3.5 ± 6.3	**−1.5 ± 5.3**	2.3 ± 4.9
Gait Breeds Gait*Breeds	**p < 0.001**		**p < 0.001**		**p = 0.007**		**n.s**.	
	**p < 0.001**		**p < 0.001**		**p < 0.001**		**p < 0.001**	
	**p < 0.001**		**p < 0.001**		**p < 0.001**		**p < 0.001**	
Post-Hoc-tests	**1** ^(2*** 3*** 4***)^		**1** ^(2*** 3*** 4***)^		**1** ^(2*** 3*** 4***)^		**1** ^(2*** 3*** 4***)^	
	**2** ^(1*** 4***)^		**2** ^(1*** 3** 4***)^		**2** ^(1*** 3*** 4***)^		**2** ^(1*** 4***)^	
	**3** ^(1*** 4***)^		**3** ^(1*** 2** 4***)^		**3** ^(1*** 2*** 4***)^		**3** ^(1*** 4***)^	
	**4** ^(1*** 3*** 4***)^		**4** ^(1*** 3*** 4***)^		**4** ^(1*** 3*** 4***)^		**4** ^(1*** 3*** 4***)^	
***Stifle***								
Wh_w (1)	**−6 ± 5.1**	−4 ± 5.1	**−5.1 ± 5.6**	−6.5 ± 5.4	**−5 ± 4.3**	−0.5 ± 3.5	**1.2 ± 3.9**	−1.4 ± 4.3
Wh_t (1)	**−7 ± 5.3**	−7.3 ± 3.7	**−7.7 ± 3.9**	−8.7 ± 3.6	**−9 ± 3.4**	−3.5 ± 3	**1 ± 3.1**	−1.8 ± 2.8
FB_w (2)	**−7.3 ± 6.1**	−5.3 ± 8.2	**−3.2 ± 8.8**	−4.6 ± 8.2	**−4.4 ± 4.9**	−1.8 ± 5.3	**−2.5 ± 7.3**	−5.6 ± 7.1
FB_ t (2)	**−6.3 ± 4.9**	−3.4 ± 6.5	**−2.1 ± 7.5**	−4.6 ± 7.8	**−8 ± 6**	−4 ± 5.2	**3.2 ± 6.6**	−2.2 ± 7.6
Be_w (4)	**−2.3 ± 6**	−4.6 ± 6.9	**−7.1 ± 4.9**	−8.8 ± 3.6	**−10 ± 3.1**	−8.6 ± 4.9	**−6.9 ± 7.7**	−3.3 ± 5.9
Be_t (4)	**−4.0 ± 7.7**	−2.5 ± 6.8	**−1.3 ± 5.6**	−2.5 ± 4.4	**−4.6 ± 5.5**	−1.1 ± 6	**2.9 ± 4.1**	2 ± 5.5
Gait Breeds Gait*Breeds	**n.s**		**n.s**.		**n.s**.		**p < 0.001**	
	**p = 0.032**		**n.s**.		**n.s**.		**n.s**.	
	**n.s**.		**p = 0.032**		**p < 0.001**		**p < 0.001**	
Post-Hoc-tests	**1** ^**(n.s)**^		**1** ^**(n.s)**^		1^(n.s)^		**1** ^**(n.s)**^	
	**2** ^(4*)^		**2** ^**(n.s)**^		2^(n.s)^		**2** ^**(n.s)**^	
	**-------**		**-------**		-------		**-------**	
	**4** ^(2*)^		**4** ^**(n.s)**^		4^(n.s)^		**4** ^**(n.s)**^	
***Femur***								
Wh_w (1)	4.7 ± 6.9	3.1 ± 5.9	2.8 ± 5.3	2.2 ± 4.9	1.8 ± 6.7	−0.5 ± 6.1	−0.3 ± 5.8	2.3 ± 5.7
Wh_t (1)	7.4 ± 6.7	6.3 ± 6.4	5.6 ± 5.8	4.4 ± 5	2.7 ± 5.9	3.8 ± 7.6	3.2 ± 5.1	5.3 ± 4.2
FB_w (2)	8.8 ± 4.2	18.1 ± 4.9	25.4 ± 6.6	34.3 ± 7.9	27.3 ± 7.2	15.8 ± 7	8.1 ± 6.7	6.2 ± 5.4
FB_t (2)	8.4 ± 8.2	10.8 ± 7.2	16.2 ± 5.9	26.5 ± 4.8	37.4 ± 6.3	30.1 ± 6.6	15.5 ± 8.8	11.2 ± 10.1
Ma_w (3)	8.5 ± 8.9	9.4 ± 9	12.5 ± 8.6	17.9 ± 10.8	19.8 ± 8	12.1 ± 9.4	8.1 ± 8.5	8.3 ± 8.5
Ma_t (3)	1.6 ± 8.6	1.1 ± 9.8	1.2 ± 8.9	1.2 ± 10.5	1.4 ± 9.3	0.7 ± 10.9	1.6 ± 9.7	3.4 ± 8.3
Be_w (4)	−10.4 ± 6.2	−6.6 ± 4.7	−5.9 ± 5.4	−11.4 ± 8.2	−14 ± 9.8	−12.8 ± 8.8	−13 ± 6.1	−12.8 ± 5.5
Be_t (4)	3.6±	4.1±	4.4±	5.1±	5.2±	1.7±	1.7±	3.3±
***Tibia***								
Wh_w (1)	−4.2 ± 10	−1.7 ± 9.2	0.5 ± 9.5	2.1 ± 8.7	3 ± 7.7	2.8 ± 7.4	1.6 ± 8	−0.9 ± 9.3
Wh_t (1)	−6.1 ± 9.7	−6.4 ± 8.9	−5.0 ± 7.8	−3.3 ± 7.4	−2.2 ± 8	3 ± 6.7	2.3 ± 7.3	−3.2 ± 9
FB_w (2)	−0.8 ± 6.6	12 ± 7.8	20.2 ± 6.9	18.7 ± 5.4	7.9 ± 4.5	1.7 ± 4.7	−3.9 ± 5.1	−5.2 ± 5.4
FB_t (2)	−6.3 ± 4.9	−3.4 ± 6.5	−2.1 ± 7.5	−4.6 ± 7.8	−8 ± 6	−4 ± 5.2	3.2 ± 6.6	−2.2 ± 7.6
Be_w (4)	−3.1 ± 7.7	0.6 ± 9.3	3.7 ± 9	2.2 ± 8.7	−1.3 ± 10.5	−3.9 ± 10.7	−3.2 ± 8.8	−1.7 ± 8
Be_t (4)	−4 ± 7.7	−2.5 ± 6.8	−1.3 ± 5.6	−2.5 ± 4.4	−4.6 ± 5.5	−1.1 ± 6	2.9 ± 4.1	2 ± 5.5

Mean and standard deviations of segment and joint kinematics are presented in degrees [°]. Bold areas display timepoints at which statistical tests were applied. SD: standard deviation, TD: touch-down, TO: toe-off, Wh: Whippet, FB: French Bulldog, Ma: Malinois, Be: Beagle, w- walk, t: trot, *p < 0.05, **p < 0.01, ***p < 0.001, n.s.: non-significant.

#### Segment and joint kinematics around latero-medial axes (segment protraction-retraction, joint flexion-extension; Figs 1 and 2)

At first glance, the overall shape of the trajectories in the sagittal plane appears to be fairly similar for all limb segments in all of the four different breeds and regardless of the measurement technique (fluoroscopy-based or marker-based). The pelvis shows a pronounced biphasic pattern (“pitch”), the hock joint exhibits a short period of flexion at the beginning of the stance phase and the limb is continuously retracted until toe-off (hip) or even into the swing phase (lower leg, foot). The Beagle’s pelvis is significantly (p < 0.001) less inclined than that of the other breeds 15° less than that of the French bulldog and Whippet and 23° less than that of the Malinois. The latter three’s pelvis behaves in much the same way, and this similarity is most pronounced in the trot. Only at TD were significant differences in pelvis tilt found between Whippets and Malinois (p < 0.01). Breed was highly significant at all analyzed timepoints (p < 0.001). Interactions between gait and breed were found not to be significant at TO and midswing. When it comes to retraction, the hind limb kinematics of the French bulldog deviate clearly from the hind limb kinematics of the other breeds. The amplitude of the femur is lower, mainly because its angle of retraction is up to 20° smaller. Its touch down position in the trot is steeper although it has a higher duty factor. The foot is less retracted in the French bulldog and the Whippet than in the Malinois and the Beagle.

**Figure 1:**
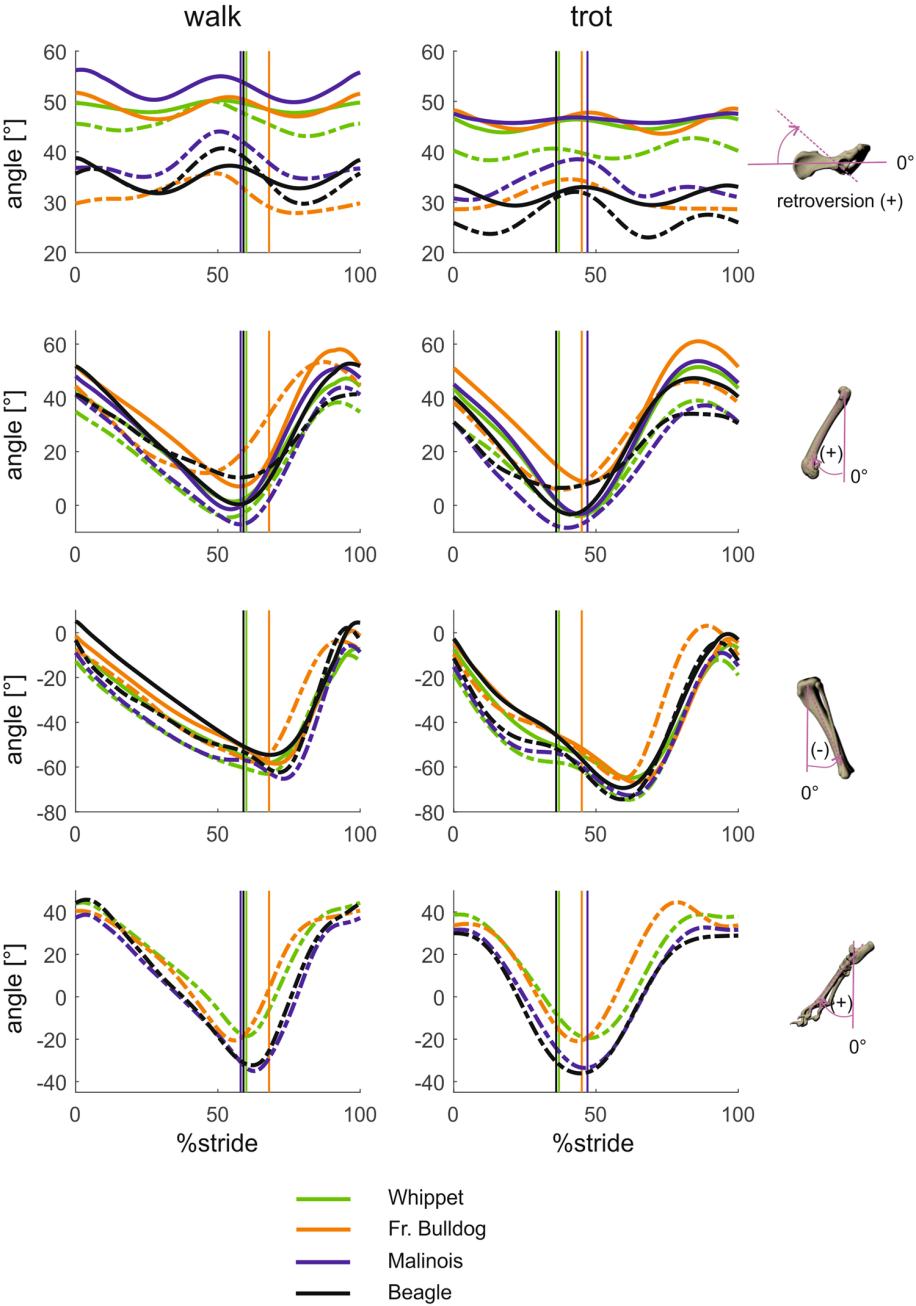
Mean curves for segment angle for each segment of the hindlimb around the lateromedial axis throughout a stride cycle at a walk (left) and trot (right). Green curves represent Whippets, orange French bulldogs, blue Malinois, and black Beagle. Solid lines represent data obtained from Scientific Rotoscoping, while dashed lines represent data obtained from marker-based data. For all graphs, values of 0 and 100 indicate TD and solid vertical lines indicate TO. 0° indicates that the segment is parallel to the vertical axis, positive values indicate protraction, and negative values indicate retraction. For the pelvis positive values indicate retroversion, and negative values indicate anteversion.

**Figure 2:**
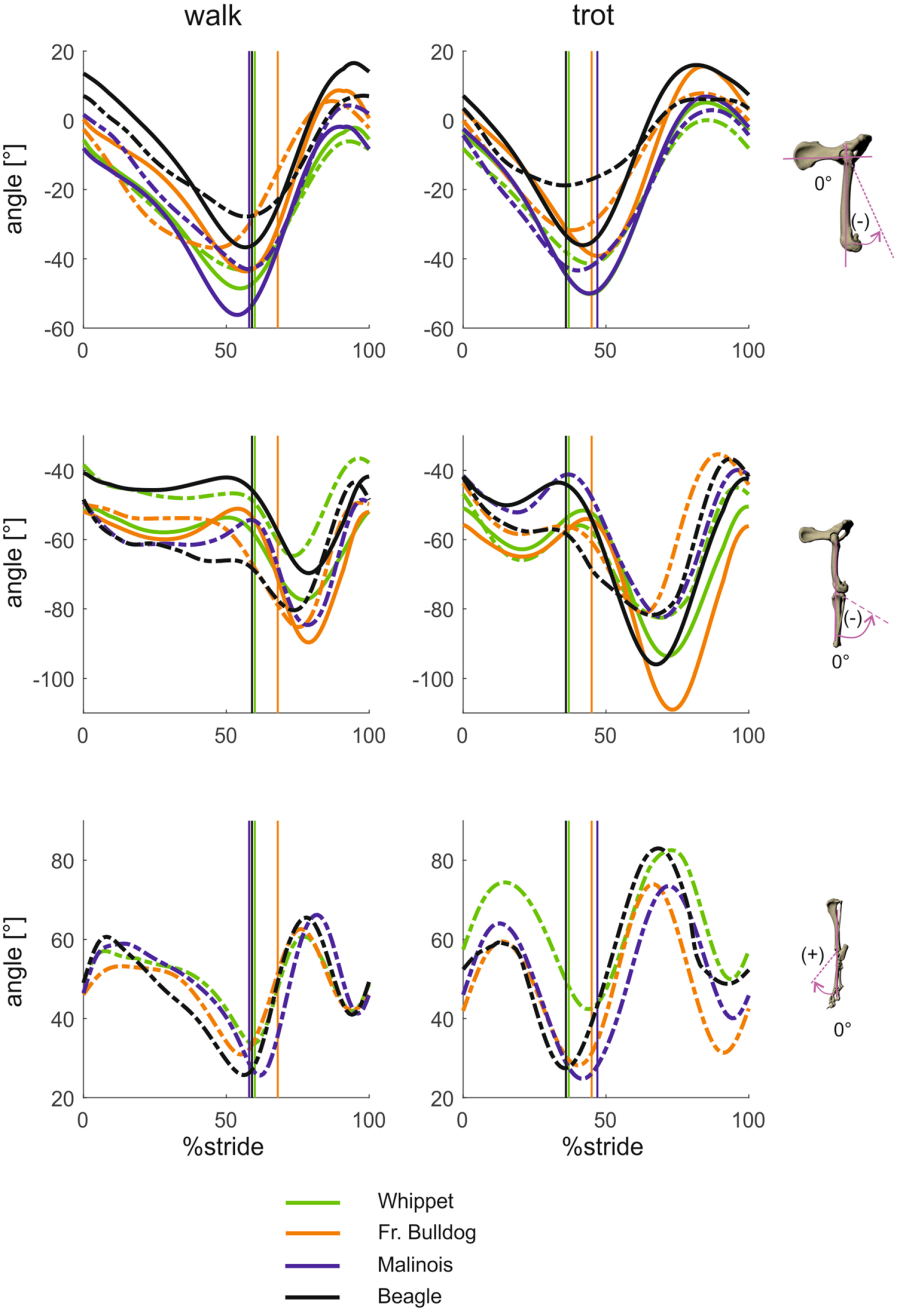
Mean curves for joint angles of the hindlimb around the joint’s lateromedial axis throughout a stride cycle at a walk (left) and trot (right). Green curves represent Whippets, orange French bulldogs, blue Malinois, and black Beagle. Solid lines represent data obtained from Scientific Rotoscoping, while dashed lines represent data obtained from a marker-based system. For all graphs, values of 0 and 100 indicate TD and solid vertical lines indicate TO. 0° indicates the reference pose of the bone’s marionet, positive values indicate joint flexion, and negative values indicate joint extension.

As usual, marker-based data are more reliable the more distally they are measured. They are close to being non-informative or false for the pelvis, deviate significantly in the amplitudes and timing of events for the femur (in the French bulldog, for example, there was a 37° difference in amplitude, and the start of protraction was 12% earlier), and are reliable distal to the stifle.

Flexion of the hock joint starts late in the swing phase and continues into the first quarter of the stance phase in all breeds but the French bulldog, where it lasts until midstance. Flexion of the stifle joint starts earlier than that of the hock joint in the last quarter of the swing phase and continues in all breeds for about the first two thirds of the stance phase, after which extension begins. Extension of the hip joint starts in the last third of the swing phase and ends shortly before toe-off.

In all major hind limb joint curves are shifted up to 10°–15° between the breeds. The French bulldog often starts with the lowest values at touch down, but the overall pattern remains the same. Effective angular movements (the difference between angles at touch down and toe-off) are as follows: hip joint during walk/trot: Malinois 47°/48°, Whippet 41°/43°, Beagle 41°/40° and French bulldog 36°/41°; stifle joint during walk/trot: Whippet 5°/4°, Beagle 4°/2° and French bulldog 14°/1°. The high effective angular movement seen in the French bulldog’s walk comes from a delayed toe-off. It is also interesting to note that the turning points, especially during the swing phase, are synchronous in the respective gait in all four breeds despite different swing phase durations. With the only exception at midstance, hip flexion-extension angles were not gait-related (p > 0.05). At TD, Beagles exhibit significantly greater hip flexion (p < 0.001) to compensate for their lower pelvic retroversion. At TD and at midstance, Malinios and Whippets exhibit similar hip extensions, as do French bulldogs and Beagles at TO and at midswing (p > 0.05).

Excepting at TD, stifle flexion-extension angles were gait related (p < 0.001). Interactions between gait and breeds were not significant at TD and midstance. At TD and midstance, the stifle joint is significantly less flexed in Beagles (p < 0.001). At TO all three breeds displayed different stifle flexion angles (p < 0.01). Finally, at midswing French bulldogs exhibited significantly greater stifle flexion (p < 0.001).

The protraction-retraction amplitudes for the hip and stifle joint determined by fluoroscopy/SR differed substantially from those determined using external markers.

Mean values and standard deviation of segmental and joint kinematics at specific timepoints based on scientific rotoscoping can be found together with the results of the statistical tests in Table 1. Marker-based data can be found in the supplementary (Tables S1 to S8).

#### Segment and joint kinematics around craniocaudal axes (segment and joint abduction-adduction; Figs 3 and 4)

Pelvic movement (“roll”) is biphasic and most strongly expressed in the French bulldog, where the maximum amplitude is 16° (walk) and 19.5° (trot). In the Whippet, the pelvis is held strictly in place, exhibiting a lateral excursion of less than 3°. A pairwise comparison reveals that at TD, pelvic roll deviates significantly in Whippets (p < 0.001). At midstance significant differences exist between Malinois and Whippets (p < 0.05) and between Malinois and French bulldogs (p < 0.05). At TO and midswing there were mostly highly significant differences in angles across the breeds (p < 0.001).

**Figure 3:**
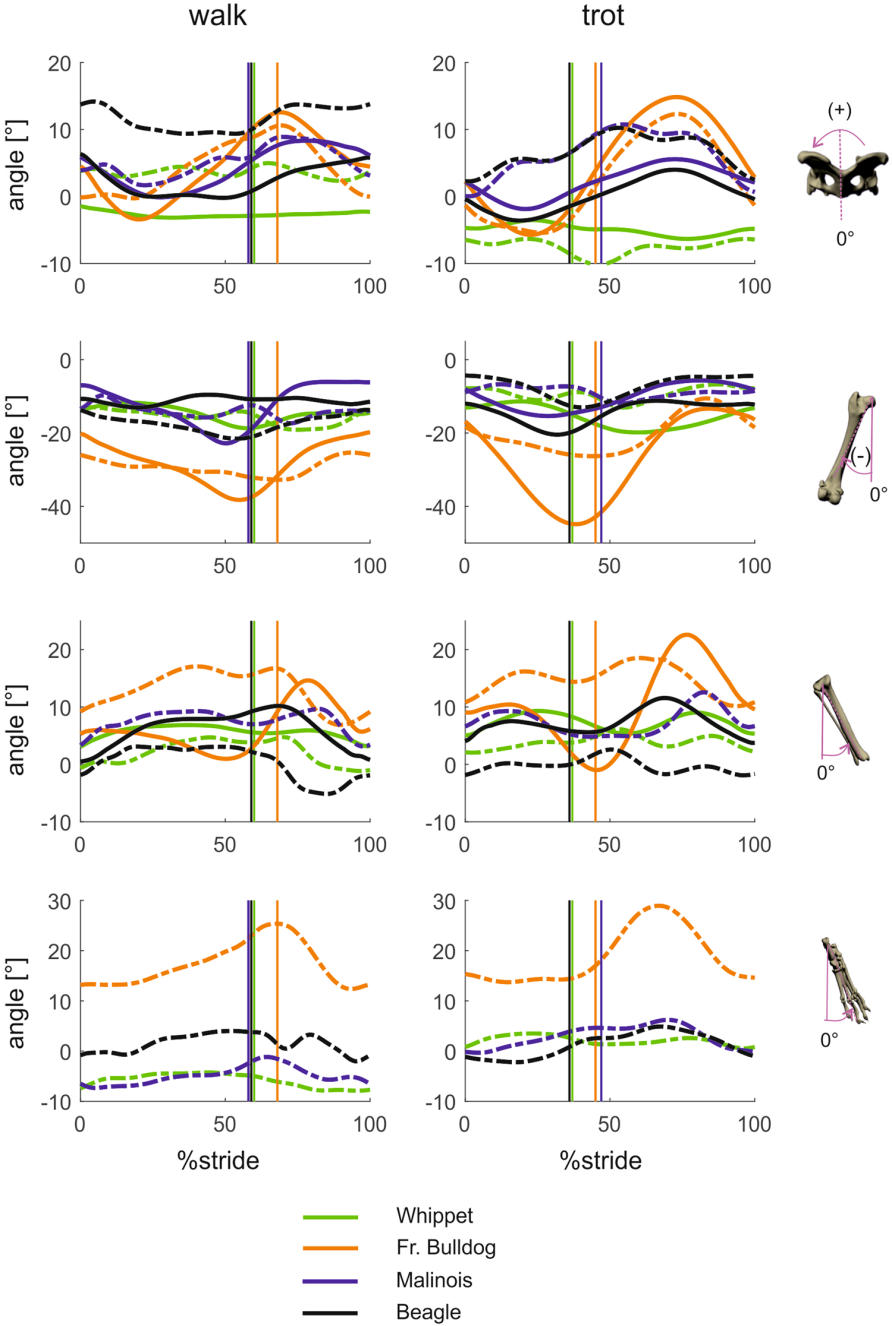
Mean curves for segment angle for each segment of the hindlimb around the craniocaudal axis throughout a stride cycle at a walk (left) and trot (right). Green curves represent Whippets, orange French bulldogs, blue Malinois, and black Beagle. Solid lines represent data obtained from Scientific Rotoscoping, while dashed lines represent data obtained from marker-based system. For all graphs, values of 0 and 100 indicate TD and solid vertical lines indicate TO. 0° indicates that the segment is parallel to the vertical axis, positive values indicate adduction, and negative values indicate abduction. For the pelvis positive values indicate roll motions towards left, and negative values indicate roll motions towards right.

**Figure 4:**
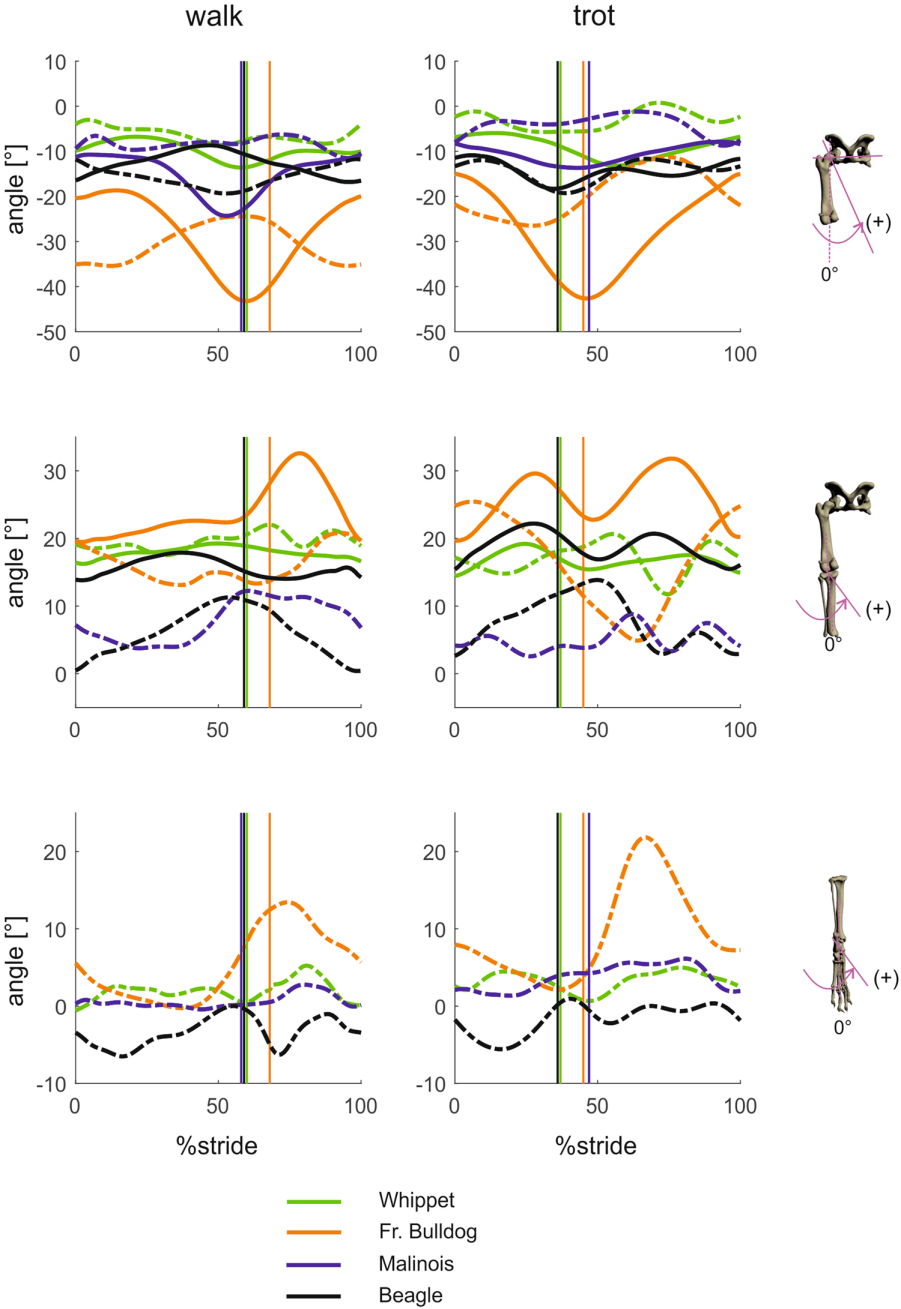
Mean curves for joint angles of the hindlimb around the joint’s craniocaudal axis throughout a stride cycle at a walk (left) and trot (right). Green curves represent Whippets, orange French bulldogs, blue Malinois, and black Beagle. Solid lines represent data obtained from Scientific Rotoscoping, while dashed lines represent data from a marker-based system. For all graphs, values of 0 and 100 indicate TD and solid vertical lines indicate TO. 0° indicates the reference pose of the bone’s marionet, positive values indicate adduction, and negative values indicate abduction.

Femur excursions around craniocaudal axis are basically biphasic. Their amplitude during the stance phase increases from the Whippet (walk 6°/trot 3°) to the Beagle (2°/8°) to the Malinois (14°/7°) and then drastically to the French bulldog. There is a real quantitative leap between the first three breeds, in which excursions remain mainly under 10°, and the French bulldog, which displays around 18° (walk) and 27° (trot) of femoral abduction during stance. The femur starts to be abducted at touch down and this movement continues until close to toe-off. It is reversed earliest in the Beagle (walk) and the French bulldog, which shows a later toe-off in normalized curves than the other breeds. The trotting Whippet deviates from this pattern, in slightly adducting the femur until the last third of the stance phase before reversing to abduction (Fig. 3). At all measured timepoints was hip abduction in French bulldogs is significantly higher (p < 0.001, except between Beagle and French bulldog at TD, p < 0.05).

At TD and TO, stifle abduction-adduction angles are not gait-related (p > 0.05), and interactions between gait and breed were not found to be significant either (p > 0.05). During all analyzed stride events, stifle adduction was significantly greater in the French bulldog (p > 0.001) and did not differ significantly between Whippets and Beagles.

The tibia is slightly adducted in the Beagle and even less adducted in the Whippet during the stance phase, and the increase in adduction is insignificant during the trot. Here too, the French bulldog deviates from the other breeds by abducting the tibia until the last quarter of the stance phase, then adducting it rapidly throughout toe-off until about 40% of the swing phase, when abduction begins again. During the trot, abduction during the stance phase continues until toe-off, when it is followed by strong adduction until midswing.

During the stance phase, the foot is slightly adducted in the Whippet and strongly adducted in the French bulldog, with the other two breeds somewhere in between, but closer to the Whippet.

Like the stifle and the hip joint, the hock joint offers an inhomogeneous picture of kinematic trajectories. All breeds except the French bulldog start stance in a close to zero position in the walk and the trot. While the Beagle first abducts before reverting to adduction from about a third of the stance phase until toe-off, followed by rapid abduction, then adduction again, and this during both the walk and the trot, the Malinois shows almost no movement in the hock joint. In contrast, the Whippet first adducts, abducts slightly at toe-off, then adducts again. The French bulldog starts in an abducted position, adducts to an almost zero position in the later stance phase, and begins a weak (walk) but strong (trot) abduction in early swing. During the remainder of the swing phase it adducts.

Stifle abduction/adduction is restricted to about 3° in the Whippet and 5° in the Beagle but reaches over 10° in the French bulldog. Trajectories are mildly biphasic during the walk and pronouncedly so during the trot, always starting with adduction. As expected, amplitudes increase from the Whippet to the French bulldog. We have no SR data for the Malinois and are reluctant to comment on the marker data for this breed.

Hip joint abduction is slight at first and increases strongly (French bulldog) or less strongly (Whippet) until late stance (walk) or toe-off (trot). Amplitudes during the stance phase are walk 22° and trot 37° (French bulldog), 13/5 (Malinois) and 6/3 (Whippet). During the swing phase, the direction of stance movement is simply reversed. The Beagle deviates here inasmuch as it adducts for most of the duration of stance.

Skin marker data not only document different trajectories and smaller amplitudes than fluoroscopic data, but are actually contradictory when it comes to the proximal joints. A comparison of the curves of the hip and stifle joints of the French bulldog, for example, reveals that skin markers have them moving in opposite directions and fail to capture their biphasic character.

Mean values and standard deviation of segmental and joint kinematics at specific timepoints based on scientific rotoscoping can be found together with the results of the statistical tests in Table 2. Marker-based data can be found in the supplementary (Tables S1 to S8).

#### Segment and joint kinematics around distal-proximal axes (pelvic lateral rotations, segment internal-external rotations and joint long axis rotation; Figs 5 and 6)

In Whippets and Malinois, the pelvis shows no or almost no rotation (“yaw”) in the stance phase either during the walk or during the trot. Movements around distal-proximal axes are measurable in Beagles (walk 5°/trot 5°) and extensive in French bulldogs (9°/12°).

**Figure 5:**
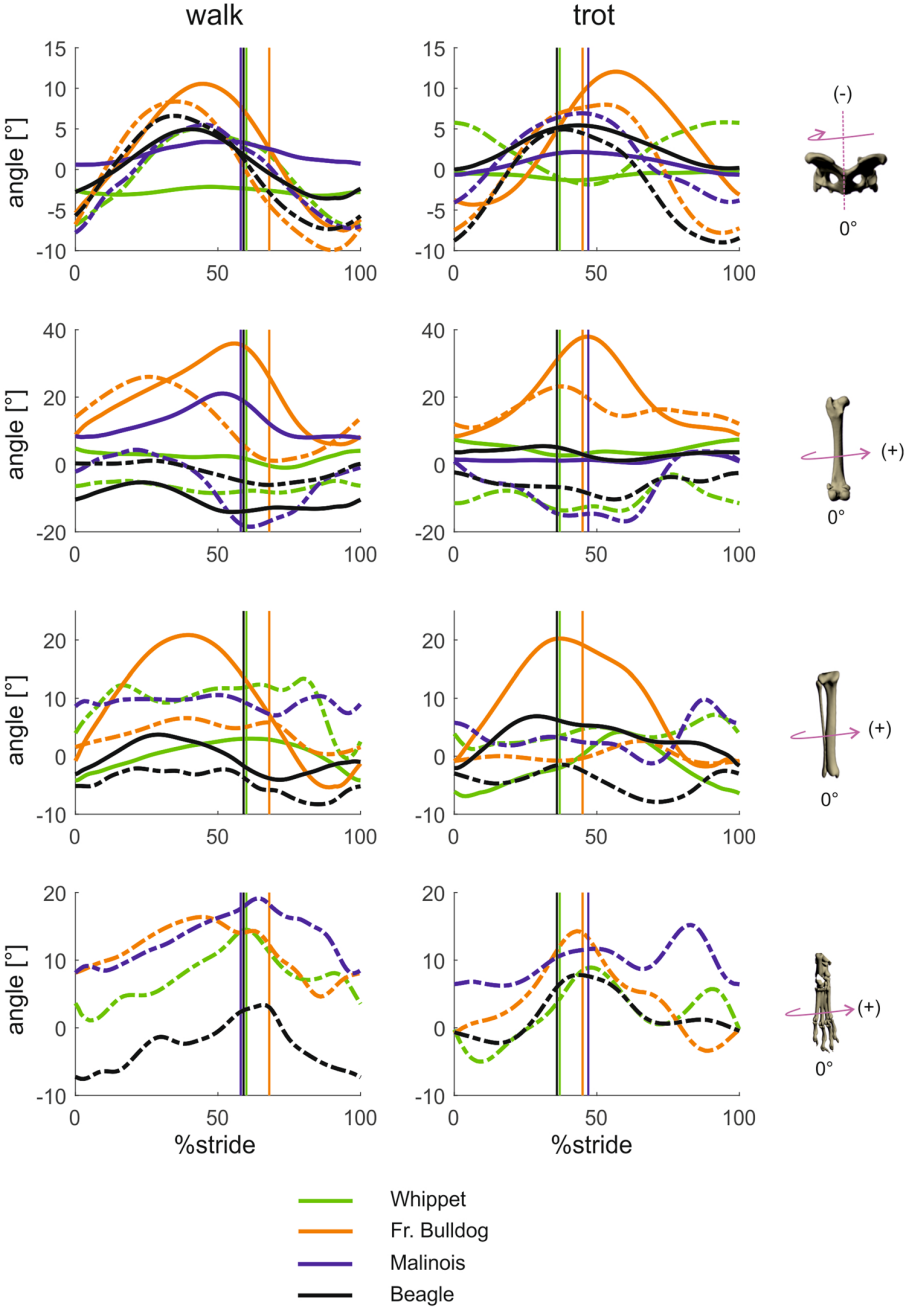
Mean curves for segment angle for each segment of the hindlimb around the distal-proximal axis throughout a stride cycle at a walk (left) and trot (right). Green curves represent Whippets, orange French bulldogs, blue Malinois, and black Beagle. Solid lines represent data obtained from Scientific Rotoscoping, while dashed lines represent data from marker-based data. For all graphs, values of 0 and 100 indicate TD and solid vertical lines indicate TO. 0° indicates that the segment is in alignment with the laboratory frame (global frame), positive values indicate external rotation, and negative values indicate internal rotation. For the pelvis positive values indicate yaw motions towards left, and negative values indicate yaw motions towards right.

**Figure 6:**
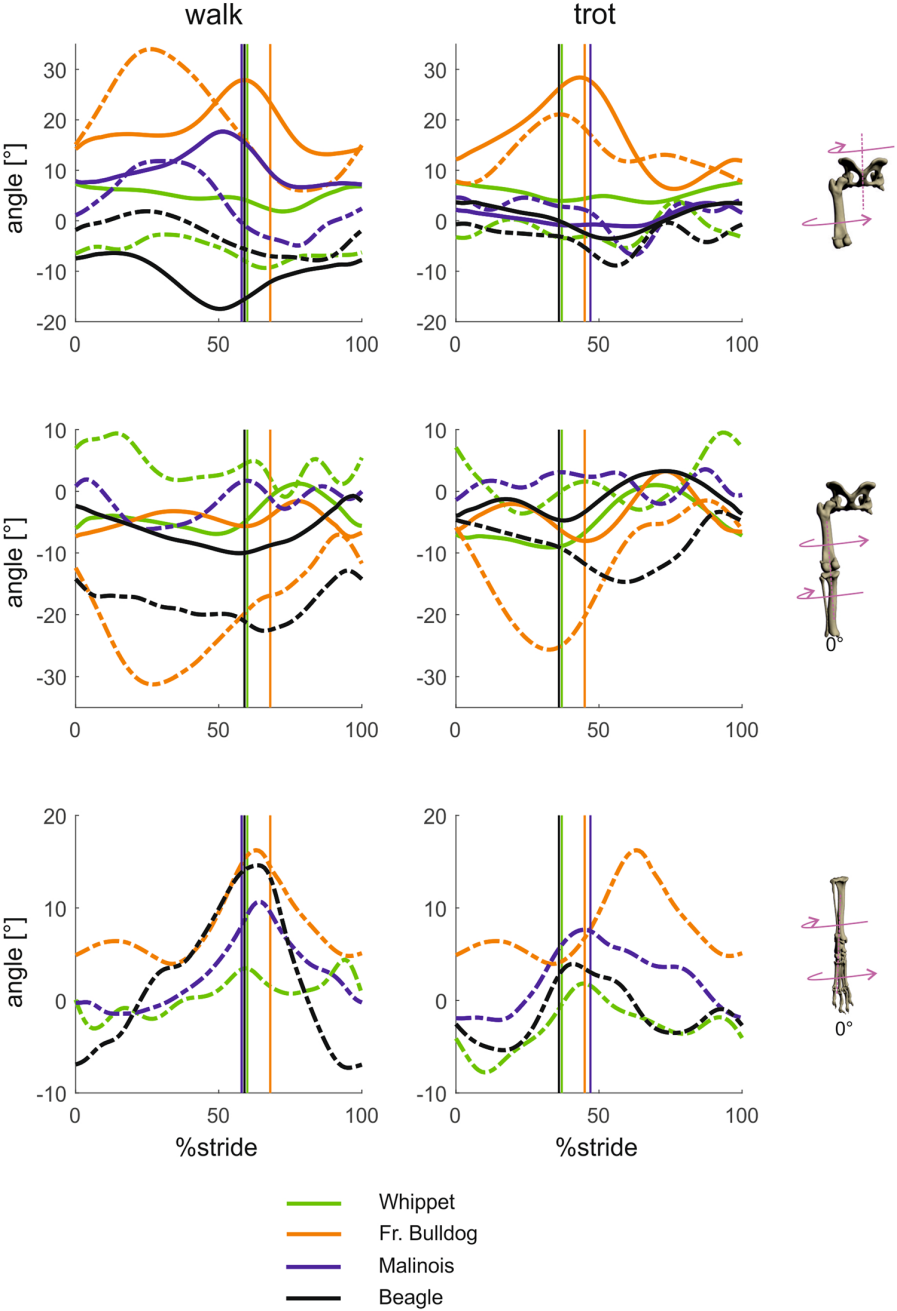
Mean curves for joint angles of the hindlimb around the joint’s distal-proximal axis throughout a stride cycle at a walk (left) and trot (right). Green curves represent Whippets, orange French bulldogs, blue Malinois, and black Beagle. Solid lines represent data obtained from Scientific Rotoscoping, while dashed lines represent data from a marker-based system. For all graphs, values of 0 and 100 indicate TD and solid vertical lines indicate TO. 0° indicates the reference pose of the bone’s marionet, positive and negative values indicate torsion.

French bulldogs exhibit significantly different pelvic lateral rotation angles (p < 0.01) at touchdown, as do Whippets at midstance (p < 0.001). At TO, the only non-significant difference in lateral rotation was between Beagles and Malinois. At midswing, significant differences were found only between Whippets and French bulldogs (p < 0.01).

External femoral rotation during the walk: French bulldog (11°), Beagle (9°), Malinois (8°) and Whippet (3°). However, while the French bulldog intensifies external rotation during the trot (16°) (Fig. 5), the other three breeds exhibit almost no rotation, and if they do it is internal (Beagle 4°, Malinois 3°, Whippet 3°). For the lower leg, the following angles were obtained: French bulldog (walk 4°/trot 6°), Beagle (8°/1°), Bhippet (1°/2°). Values for the foot were French bulldog (walk 5°/trot 14°), Beagle (10°/7°), Malinois (9°/5°) and Whippet (3°/3°). Femoral external long axis rotation usually starts during late swing, continues throughout the stance phase and ends close to toe-off. Tibial external rotation ends earlier at about 60% of the stance phase, which means that the femur continues to rotate in the stifle joint.

At TD and midswing, French bulldogs and Malinois exhibited a non-significant difference in hip axial rotation (p > 0.05), while Whippets and Beagles displayed significantly different angles with respect to the other breeds. At TO and midstance there were significant differences between breeds (p < 0.01).

The long-axis rotation of the stifle can generally be said to be neither gait nor species-related. Post hoc tests only find significant differences between French bulldog and Beagle at TD (p > 0.027).

The curve of the lower leg and foot, especially of the French bulldog, lines the strongest external rotation, but this is simply a lock-in phenomenon (Fig. 5) reflecting the way in which the external rotation of the foot with respect to the body axis is translated to the hip joint.

Long-axis rotation in the hock joint is found in all breeds and gaits except for the Whippet during the walk. The hock joint is rotated externally for most or all of the stance phase to the following extent: French bulldog (walk 11°/trot 2°), Beagle (7°/5°), Malinois (7°/10°), Whippet (3°/4°). Stifle joint’s long axis rotation curves are biphasic in the French bulldog and the Whippet but monophasic in the walking Beagle (no SR data available for Malinois). In the latter, an internal rotation of 8° occurs during the stance phase of the walk, while in the French bulldog and the Whippet, and the trotting Beagle, the stance phase starts with a slight external rotation in the first quarter (Whippet) or first half, followed by a counter-rotation which lasts until right before toe-off, followed by another external rotation until roughly 40% of the swing phase. Finally, the hip joint undergoes an 11° (walk) and 10° (trot) rotation in the French bulldog, a 9°/2° rotation in the Malinois, a 9°/2° rotation in the Beagle and a 3°/3° rotation in the Whippet.

It is interesting to note with regard to long-axis rotation or torsion measurements that external anatomical markers may lead to misleading results when it comes to the proximal joints and segments.

Mean values and standard deviation of segmental and joint kinematics at specific timepoints based on scientific rotoscoping can be found together with the results of the statistical tests in Table 3. Marker-based data can be found in the supplementary (Tables S1 to S8).

## Discussion

In a comparison of the four breeds, the French bulldog is set apart by limb-segment movements around craniocaudal axes (yaw) and by long axis rotation (roll). Femoral long axis rotation leads to lower limb reduction in the unloaded limb or with a fixed ground contact to pelvic displacement together with yaw and roll motions. French bulldogs translate extensive femoral long axis rotation (>30°) into a pronounced tilting and medial displacement of the pelvis even in the trot in order to compensate for the highly abducted position of the hindlimb from the beginning of stance (Fig. 3). This unusual hindlimb trajectory is caused by the animal’s barrel-shaped trunk. Interestingly, and contrary to our assumptions, stifle torsion in the French bulldog was not different from the other breeds we analyzed (see movements around distal-proximal axes). French bulldogs also show the largest pelvic rotations.

Our data on pelvic motions obtained from Beagles correspond to only available detailed 3D-measurements in the intervertebral from S1/L7 to L2/L1 published by Wachs *et al*.^20^. They found small lumbar intervertebral joint motions in both walking and running (<6°). These amplitudes decrease cranially. In our study Beagles displayed a significant lower pelvis retroversion than the other breeds analyzed in our study. But their femoral retraction/protraction is fair similar to those displayed by the Malinois and the Whippet. The consequence is that Beagles exhibit significantly greater hip flexion at TD, and then lower hip extension during stance.

Headrick^33^ and Headrick *et al*.^9^ were pioneers in describing the 3D kinematics and inverse dynamics of the canine hind limb. Kim *et al*.^11^ provided a groundbreaking description of the 3D kinematics of the stifle joint based on single plane fluoroscopy. To our knowledge, however, the present study is the first high-precision 3D *in vivo* investigation into the kinematics of the entire canine pelvic limb during a walk and a trot. It uses biplanar, high-frequency fluoroscopy in combination with a high-frequency 3D optoelectric system (Vicon) and a newly developed marker setup, and also utilizes “Scientific Rotoscoping”^34^ (the “3D-2D registration process” described, for example, by^11,25^). The present study shows that only this very time-consuming method permits a convincing analysis of 3D kinematics in dogs without implanted bone markers.

### Remarks on different methods

Before we compare our findings with earlier data, it must be made clear that the different methods used to obtain data drastically influence the results. The movement of skin markers relative to the underlying bone has already been discussed as a major source of error in kinematic analyses (^23,35–38^). The possible error already significant in sagittal plane kinematics, especially with regard to the proximal joints, is beyond tolerance in 3D kinematics. Amplitudes and the timing of trajectories are different when recorded using skin markers. A comparison of the abduction/adduction or long axis rotation of the hip and stifle joint in the French bulldog even reveals that markers show them moving in opposite directions. We believe that motion capture was tested to or beyond its limits by our French bulldogs because the distance between markers was sometimes less than two centimeters due to the animals’ small size, and two centimeters is the minimum distance at which our camera setup is able to accurately differentiate them. Six cameras may therefore not have been enough to capture accurate data, but as setup modification was not allowed in this clinical lab we were unable to add any extra.

Headrick’s kinematic data on six hound-type dogs were recorded at 60 Hz using a 4-camera 3D motion capture system. Fu *et al*.^29^ captured data on 6 mixed breed dogs at 200 Hz using 8 cameras, Kim *et al*.^28^ at 180 Hz with 6 cameras (Labrador Retriever (n = 4), Golden Retriever (1) and Greyhound (1)). A frequency of 60 Hz results in 10 frames at best for a dog trotting slowly, and fewer than 6 cameras will leave too many markers hidden, especially when it comes to medial movements. According to our own long experience of 3D motion capture systems (both Qualisys and Vicon), calculating long axis rotation on the basis of single markers is particularly problematic, and even the T-markers we used did not offer convincing results.

The error inherent in SR was estimated to be the mean of standard deviation during a single stride. In all analyzed cases, average variation did not exceed ±3°. This error is close to the one computed in rats with the next gold standard (±2°, marker based XROMM, Bonnan *et al*. 2016). As a general rule, the greatest variability was seen in connection with the French bulldog. Variability was normally the lowest in the estimation of protraction-retraction angles, where it remained below ±1° (except in French bulldogs). It was the highest, generally speaking, when it came to internal-external rotations/torsions, correlating with the difficulty involved in matching the shadow of the bones at each degree of freedom.

Malinois display the higher interindividual variation in motions around distal-proximal axes. This may indicate, as stated in the methods, that results obtained when the X-ray beams are on an incline are less informative due to the more difficult SR assessment.

The single plane fluoroscopic analysis described in^11^ was carried out at 60 Hz on 6 Labrador Retrievers, that described in^25^ at 30 Hz on 5 mixed breed and 5 different purebred dogs.

### Movements around latero-medial axes

We hesitate to place too much emphasis on the differences in the kinematic data obtained by the studies mentioned above as they could well be due to the varying constraints of each method. Sagittal plane data were presented and discussed at length in our study on 327 dogs of 32 different breeds^8^, with the key result being that intra-breed variation often exceeds that between breeds, e.g. variation within Great Danes is higher than that between the mean values obtained for this breed and those obtained for Dachshunds^39^. The data presented here, too, reveal homogeneity in the trajectories of limb segments and joints during retraction and protraction and are concurrent with the fluoroscopy data obtained by^11^ and our own biplanar data^8^. They also confirm the dominant role of femur retraction in the retraction of the hind limb as a whole, as shown by effective angular movements of 40° set against stifle joint angles which remain in the single-digit range. Hip joint excursion contributes to progression, while as in the stifle joint higher maximum than effective angular movement shows the degree of non-progressive, vertical modulating work.

### Movements around craniocaudal axes

During the stance phase, the foot and lower leg are slightly adducted in the Beagle and the Whippet. In the French bulldog the foot is strongly adducted but the tibia is abducted until the last quarter of the stance phase, followed by a rapid adduction throughout toe-off. Based on biplanar X-ray fluoroscopy of 5 healthy foxhounds recorded at 250 frames/s, Tashman *et al*.^27^ describe the same pattern as in the Beagle, namely a slight adduction during stance in walking. Femur excursions in the frontal plane are basically biphasic. Their amplitude during the stance phase increases from the Whippet to the Beagle and Malinois and then jumps to the French bulldog, which displays maximal values of femoral abduction related to the pelvis of around 18° (walk) and 27° (trot) during stance. The femur starts to be abducted at touch down and this movement continues almost until toe-off. The extreme abduction in the French bulldog is translated throughout the limb from the tip of the toe to the pelvis. It also becomes clear from the graphs in Figs 3 and 4 that 3D optoelectric systems simply fail to accurately decipher movements around craniocaudal axes.

The picture of kinematic trajectories painted by the pelvic limb joints is an inhomogeneous one (see results). We are therefore unable to confirm the observations of Headrick *et al*.^9^ on hound dogs that the hock joint begins the stance phase in slight abduction and then remains adducted throughout the ROM, or that the stifle also begins the stance phase slightly abducted, spends the first 50% in slight adduction and the second half of the phase in slight abduction. Torres *et al*.^40^ describe a minor abduction/adduction during the walk and abduction of about 10° during stance. Except for the Beagle the hip seems to abduct throughout the stance phase in all the breeds studied so far. This is confirmed by Fu *et al*.’s description of a slight abduction in the hip joint throughout the stance phase in mixed breeds^29^. As these authors do not comment on their results at all, it is difficult to interpret their curves, especially as their Fig. 2a,b, labeled walking and trotting respectively, are in fact identical. Korvick *et al*.^26^ found that “during swing the motion includes three rotations but during stance, only flexion-extension was present”.

### Movements around distal-proximal axes

External rotation in the hock joint is found in all breeds and gaits except in the Whippet during the walk. External rotation in the stifle joint is biphasic in the French bulldog and Whippet, and in the Beagle during trot (no SR data available for Malinois). It begins in the first quarter (Whippet) or first half of the stance phase and is followed by internal rotation until right before toe-off. This in turn is followed by external rotation until roughly 40% of the swing phase, and then by internal rotation until touch down. As in the frontal plane, Tashman *et al*.^27^ describe the same pattern in the walking foxhound as in the Beagle, i.e. an internal rotation of about 8–9° during the stance phase. Hip joint rotation is strongest in the French bulldog, less pronounced in the Malinois and Beagle, and almost not measurable in the Whippet.

Contrary to our results, Headrick *et al*.^9^ observed first an internal rotation of 15° and then an external 10° rotation of the hocks. The stifle joints of the dogs in the study by Fu *et al*.^29^ rotated less than 10° externally and then rotated internally again during the stance phase. The mixed breed dogs studied by Torres *et al*.^40^ and the hound dogs in Headrick *et al*.^9^ exhibited external rotation of about 10° for the duration of the stance phase during the walk. Torres *et al*.^40^ report a biphasic pattern starting with internal rotation in dogs at walk. Headrick *et al*.^9^ found the hip to rotate internally during stance, as did Fu *et al*.^29^, whose curve indicates this rotation to be around 15°.

The movements captured by markers in our study do not precisely resemble the curves in Headrick *et al*.^9^ or Fu *et al*.^29^ in either the frontal or the transverse plane. Headrick *et al*.^9^, for example, reported internal rotation of almost 40° in the hip joint at TD, which seems to be a very large value. Our values (both SR and marker data) oscillated between approx. 10° internal rotation and 15° external rotation at walk.

**Figure 7:**
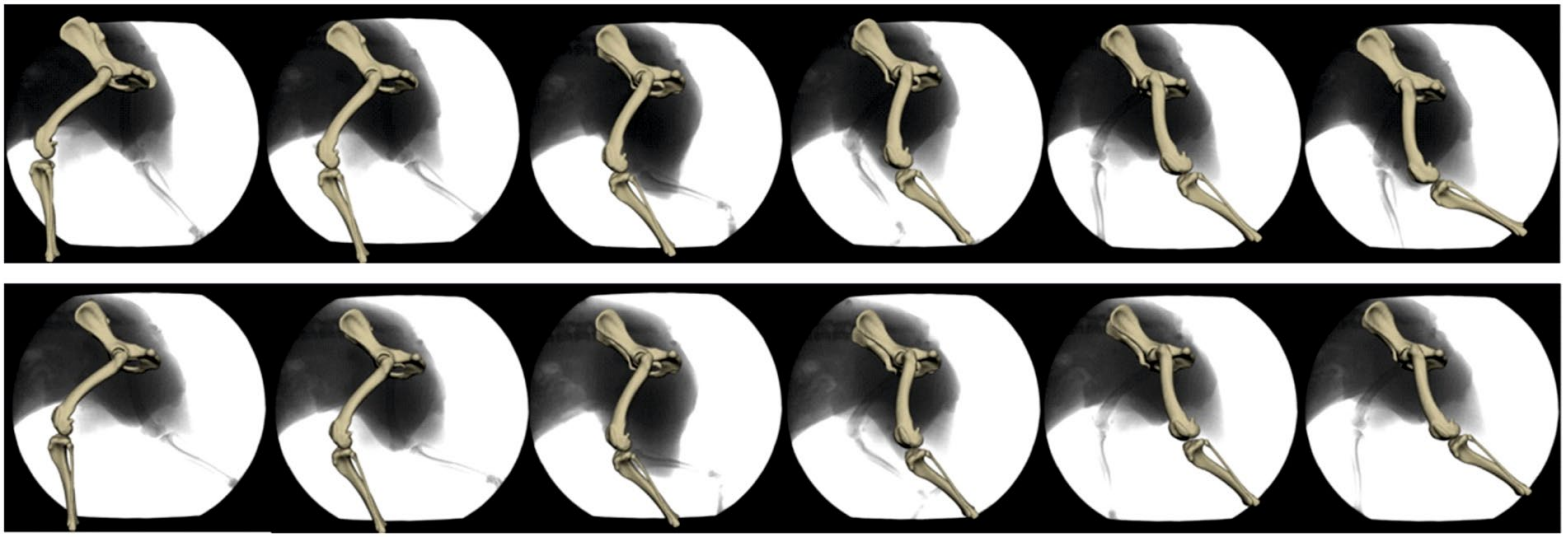
Abduction/adduction and long axis rotation during the walk (upper line) and trot (lower line) in the French bulldog. Note how the cranial surface of the femur (seen here with the patella) is turned outwards and becomes more visible and that this is more pronounced during the trot.

**Figure 8:**
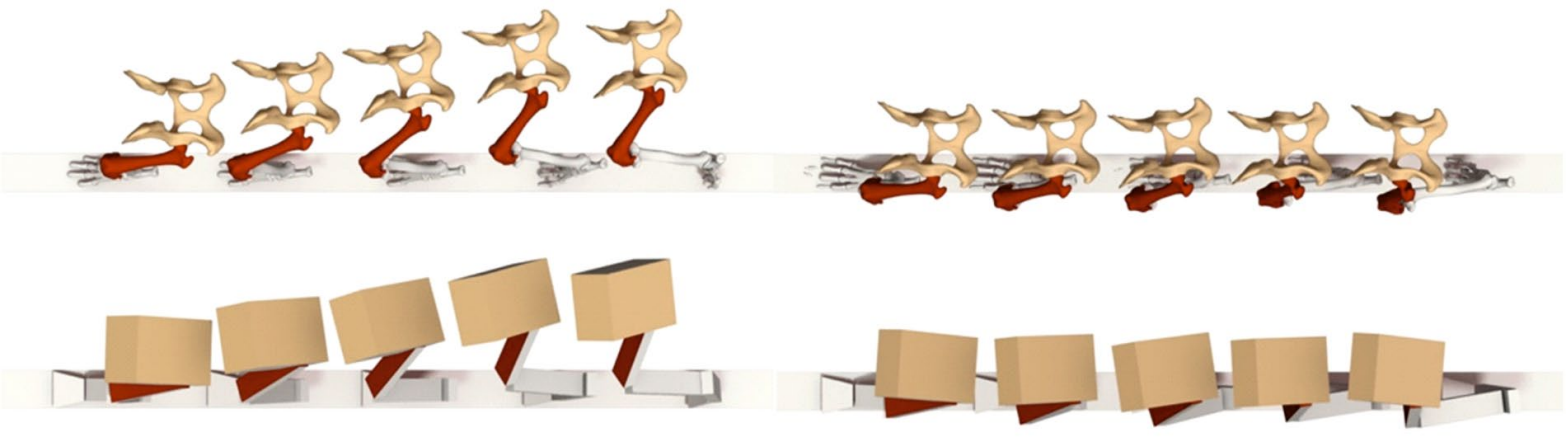
Influence of femoral and tibial rotations on pelvic movements. An abducted limb position enforces a stronger long axis rotation of the femur as in the French bulldog (left side), which can be best seen in the boxy version (below). As the hindpaw is fixed on the ground the movements are transmitted to the hip joint and hence to the pelvis leading to its pronounced tilting and medial displacement e.g. in the trot. In the Whippet the limb’ s trajectory is almost parasagittal with consequently almost no pelvic displacement. 3D- kinematics from femur and tibia are meanvalues.

Stifle torsion would be overestimated if it were to be calculated on the basis of the axial rotations of the femur and tibia alone. Not only would there be a significant difference between the French bulldog and the other breeds, but we would expect to find more than 20° stifle torsion in the French bulldog at TO. However, stifle torsion in French bulldogs was not found to be significantly different from that in Whippets or Beagles. These apparently conflicting results are explained by joint constraints. When the stifle joint is flexed, the external rotation of the femur induces a rotation of the tibia towards the midline of the body (during swing) or pelvic displacement (during stance) depending on the fixation. In a similar way, abduction of the femur induces external rotation in the tibia in the global coordinate system. Thus, breeds with more abducted hindlimbs are confined to larger external rotations in the femur in order to place the foot below the pelvis. It would be interesting to investigate whether the popliteus muscle responsible for external femoral rotation is stronger in French bulldogs than e.g. Whippets (Figs 7 and 8).

### Stifle torsion and cruciate ligaments

3D stifle kinematics were explored by Kim *et al*.^11^, whose study is the only one previous to ours to use SR, but based on single plane fluoroscopy. This makes a comparison with our study particularly pertinent. Like our dogs, the six walking and trotting Labrador Retrievers investigated by Kim *et al*. also switched from internal to external rotation of the tibia during the late swing phase. As in our study, the overall axial rotational range of motion was greater during the trot than the walk, and the range itself corresponded to that we found for all breeds except the French bulldog. Kim *et al*.^11^ were able to demonstrate *in vivo* that an increase in flexion is associated with increased internal tibial rotation during both the walk and the trot, which is confirmed by our data. They also found a slight adduction for most of the stance phase in the same range of motion as we observed in the Whippet, Beagle and Malinois but not the French bulldog. In contrast, their observation, that increased flexion is inconsistently correlated with increased abduction angulation, is not fully confirmed by our results. In fact, Kim *et al*.^11^ themselves only found this correlation in four out of six dogs.

The link between axial rotation and flexion–extension has long been known from cadaver studies as a passive restraint on canine stifle motion (the “screw-home” mechanism^41^). Axial rotation after relaxation of the lateral collateral ligament is seen as a transformation of the valgus load^42^. This mechanism has been described to cause the cruciate ligaments not only to wrap around each other, but also to spiral in on themselves. This leads to a continuous increase in tension which decelerates internal rotation^43–45^. The present study highlights great differences in long axis rotation between the breeds, and it is obvious that breeds which exhibit strong abduction during stance will show a higher long-term loading of the cruciate ligaments - especially if their body mass is high. Even with the technical capacity of the equipment at our disposal we were unable to test the effect of varus forces, which are suspected to lead to higher strain on the ACL^44^.

### The eccentric walk of the French bulldog as selection for manoeuvring

The unexpected degree of abduction and external rotation seen during the walk and even more pronouncedly during the trot sets the French bulldog apart from all the (admittedly few) other breeds studied so far. Chase *et al*.^31^ and Carrier *et al*.^32^ analyzed QTLs (quantitative trait loci) and revealed trade-offs which are evident in Portuguese water dogs, Greyhounds and Pitbulls. Speed types and strength types differ in skull and pelvis shape and in the transverse profile of their long bones (elliptical vs. round), and a difference in ribcage shape (either slim or round in cross-section) has led to a difference in the position of the limbs. 3D kinematics absolutely reflect these differences in body shape and limb position. The transverse profile of the long bones might be the crucial factor behind selection in sighthounds and Molossians in general. The long axis of the ellipse in sighthounds’ long bones corresponds with the direction of motion so mediolateral forces are low. Kinematics show that Whippets are close to perfect sagittal runners (see also Fig. 8). Bone stiffness is 1.5–2.4-fold greater in Greyhounds than in Pitbulls ^46^.

Recently, Parker *et al*.^47^ found high bootstrap support (90% or better) for a clade consisting of breeds including Boxers, various bullterriers and Molossians. Whether selection took place for bullbaiting in the 16^th^ to 18^th^ centuries until bullbaiting was banned in 1835 or whether these breeds were definitively selected in the second half of the 19^th^ century, their most characteristic movement is maneuvring. Any kind of manoeuver or turning jump is going to be easier with limbs that are already abducted. Mediolateral forces will abound, so the round transverse shape of the long bones, a broad standing posture and an obtuse-angled support triangle are of advantage. Additionally, resistance to fracturing is 2.2-fold greater in the strength type then in speed types^46^.

It is time to abandon the dogma of character or trait selection in breeding and adopt the idea that wherever selection starts, be it the skull or locomotion, it will affect other parts of the body. The ‘global’ factors that affect wide parts of the head or body^48^ are controlled by both the environment and underlying genetics^49^. Abundance of shapes is often constrained to just a few dimensions, especially when traits are genetically correlated^31,50^. In dogs, for example, the variation from brachycephalic to dolichocephalic skulls and the attendant variation in relative rostrum length determines most of the covariation pattern of the skull modules (e.g.^51,52^). This integrative view needs more support.

Since Darwin, studies into domestic animals have the potential to reveal not only a spectacular array of variation on an intraspecific level but to teach us much about diversity in general^53^. As Darwin wrote in 1859 after reflecting on the “correlation of growth”: “Breeders believe that long limbs are almost always accompanied by an elongated head.”

### Animals and methods

All experiments were approved by and carried out in strict accordance with the German Animal Welfare guidelines of the states of Thuringia (TLV) and Lower Saxony (LAVES) (Registration No. TLV Az. 22-2684-04-02-012/14, and 22-2684-04-02-009/15, LAVES 33.9-42502-04-14/1518, and 33.9-42502-04-15/1859). All dogs had to be healthy and free of orthopedic abnormalities as determined by the results of physical and orthopedic examinations.

We selected:

- five adult male Beagles belonging to a research colony based at the Small Animal Hospital of the University of Veterinary Medicine, Hannover, Germany;
- five adult Malinois (4 males/1 female) kept as police dogs by the Saxon police force;
- four adult female French bulldogs from private dog owners;
- five adult Whippets (2 males/3 females) from a private dog owner.
- Details of the dogs are listed in Table 4.

**Table 4:** Dogs and number of strides analysed for this study (SR: Scientific Rotoscoping, MC: motion capture).

Breed	Individual	Weigth [kg]	Hight at the withers [cm]	Strides SR walk	Strides SR trot	Strides MC walk	Strides MC trot
Beagle	Erwin	14.9	35	11	10	21	18
	Simon	13.8	33	9	10	15	11
	Malte	14.8	34	9	8	20	25
	Louis	16.2	38	0	0	17	22
	Spencer	19.8	42	0	0	15	24
Malinois	Zora	28.5	64	8	9	11	10
	Pike	28.5	62	10	10	11	10
	Hunter	18.6	59	10	10	10	10
	Rocky	22.4	58	9	11	9	11
	CherryLee	21.3	59	8	10	8	10
French bulldog	Queny	11	31	10	11	15	20
	MJ	9,5	30	10	9	18	19
	Chacha	10	31	9	10	16	15
	Juno	13	32	10	9	15	20
Whippet	Lilly	12	49	9	10	20	19
	Kenja	10	46	10	10	10	21
	Afrika	9	47	10	8	13	22
	Merlin	16.5	51	10	9	19	20
	Moody	13.3	50	9	10	18	16

#### 3D reconstruction of kinematics using Scientific Rotoscoping

The methodology behind the X-ray reconstruction of moving morphology (XROMM) has been described both outside our working group (e.g.^13,21,34,54^) and within it (e.g.^14,19,20,23^). We followed the workflow of markerless XROMM (Scientific Rotoscoping, SR) using Autodesk Maya™ software in combination with the XROMM Maya Tools (www.xromm.org; Brown University, Providence, US). The procedure will only be summarized briefly here. Details are given for experimental or analytical steps which are specific to our study.

Scientific Rotoscoping SR (shadow matching) has the advantage for the animal of being surgically non-invasive. The accuracy of the manual matching of the CT-based virtual bones to the biplanar X-ray shadows depends to a large extent on the contrast and sharpness of the X-ray images and on the recording perspectives selected. The latter are best when arranged orthogonally and less informative when the X-ray beams are on an incline. The experience of the investigator also plays a role. In preparation for the production of our animation, everyone involved repeated the whole manual registration procedure several times on the same X-ray video, redefining the zero position, adding additional elements and refining joint coordinate systems.

In addition, we estimated the accuracy and repeatability of the manual match by repeating the manual registration of five frames during stance (TD, 25%, 50%, 75%, TO) and two frames during swing (33% and 66%) four times. Standard deviation (SD) was computed for each degree of freedom (DOF) at each joint. Mean SD during stride is taken as an estimate of registration error for each DOF at each joint.

Dogs moved freely or on a leash on a treadmill adjusted to their individually preferred walking or trotting speed (see results). Data collection started as soon as the dogs were walking or trotting smoothly and comfortably. The animals were centered within the overlapping X-ray beams. The two beams were positioned orthogonally except in the recordings involving the Malinois, where they were arranged at angles of 63° due to these dogs’ body size. The fluoroscope settings used were 90 kVp and 70 mAs, with a sampling frequency of 250 Hz.

The biplanar C-arm fluoroscope (Neurostar™, Siemens AG, Erlangen, Germany) with 40 cm diameter image intensifiers operates with two synchronized high-speed cameras (SpeedCam™ Visario G2, Weinberger GmbH, Erlangen, Germany). Spatial resolution was set at 1536 dpi x 1024 dpi. Radiographic videos were calibrated using an acrylic calibration cuboid (200 mm × 120 mm × 120 mm) with steel spheres of ∅ 1.5 mm implanted on each surface at a distance from each other of 10 mm in each direction. The distortion of the X-ray images was corrected using a MatLab™ routine based on a recorded grid^34^.

Additionally, movement was recorded from a frontal and a lateral perspective by two high-speed normal light cameras (Speedcam Minivis E2, Weinberger GmbH, Erlangen) synchronized exactly to the frames of the fluoroscope, essentially to document touch down and toe off events.

A CT scan was performed on one specimen per breed. Segmented leg bones were obtained using the segmentation editor in Amira® (VSG, Burlington, MA, USA). As we restricted ourselves to one whole-body CT scan per breed, skeletal elements were imported into Maya™ and scaled to match the size of the animal whose motion was being recorded.

Ten steady state strides were analyzed in most cases at both a walk and a trot for each dog.

In SR, bone models are linked via virtual joints to form a hierarchical chain. Anatomical coordinate systems are implemented at each joint to measure the movement of the distally adjacent bone relative to the proximal bone, and, directly from the motion of each limb segment, the position of the segment in relation to the global coordinate system.

**Figure 9:**
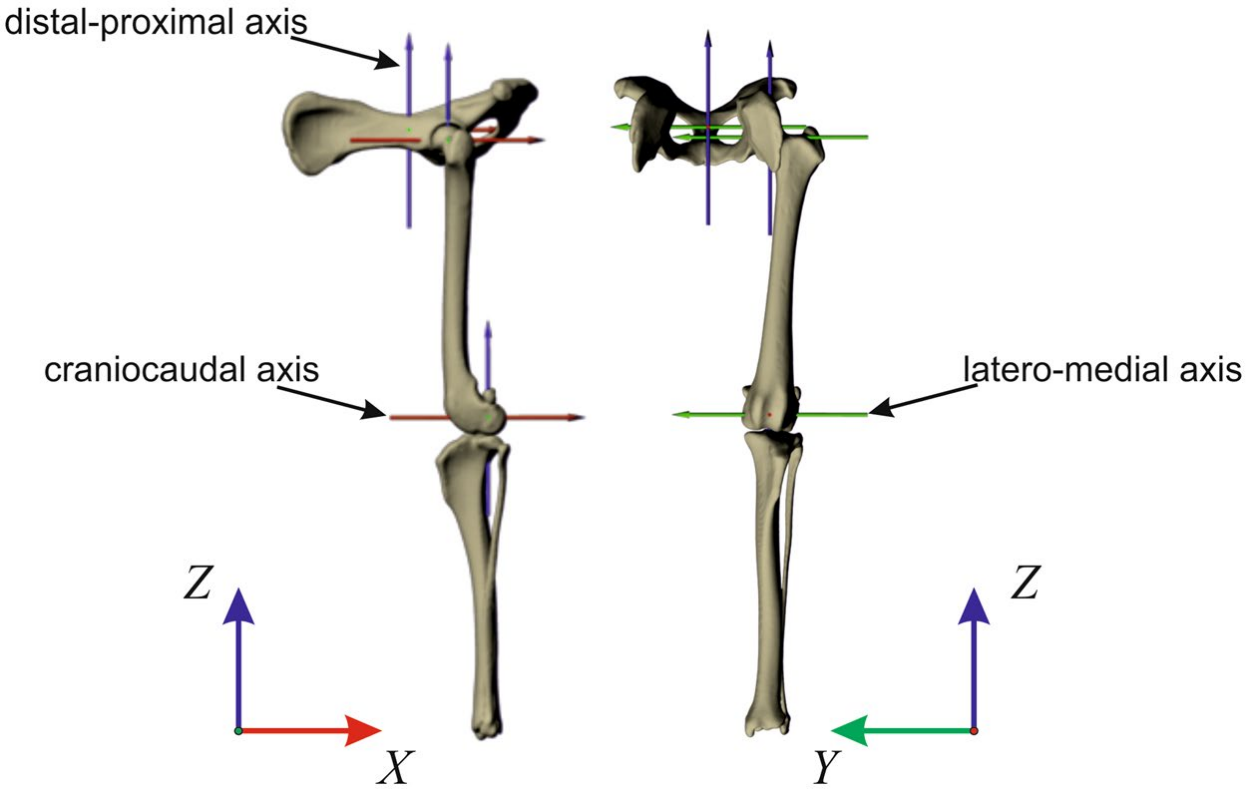
Kinematic chain (reference pose). Joint movements are measured relative to this model. Every dog has its own reference pose. However, all models were build following the same kinematic chain. Here is shown a reference pose for a Whippet. To compute segment angles (avoiding gimbal-lock), joint coordinate systems were aligned to the axes of the global coordinate system (+x points caudally, +y points medially and +z upwards).

Our 3D model consisted of three segments: pelvis, femur and tibia (which were derived from the virtual CT reconstruction). Movements were measured in relation to a reference pose. In the reference pose, all joint model coordinate systems were aligned to the axes of the global coordinate system (+x points caudally, +y points medially and +z upwards; see Fig. 9). In order to obtain anatomically meaningful data comparable to those obtained by using marker data, we used non-physiological extended reference pose for the hindlimbs (Fig. 9). Hip and knee joints were aligned in the frontal plane. The tibia was vertically oriented (positive z). In the sagittal plane, both femur and tibia were aligned to the vertical axis (z). 3D kinematics were obtained by using the sequence y, x, z, (i.e. pro/retraction, ad/abduction, axial rotation), which represents the same anatomical sequence as the one used to compute marker data (see next section).

For the model reference pose, coordinate systems were placed (1) middle of the pelvis to measure absolute motion of the pelvis related to global coordinate system (in the sagittal plane the girdle was oriented to have iliac crest and the middle of ischiadic tuberosity at the same height), (2) in the hip joint to measure femoral movement relative to the pelvis and femoral absolute motion relative to the global coordinate system (3) in the knee joint (midway between the centers of the lateral and medial condyles) to measure tibial movement relative to the femur and absolute tibial motion relative to the global coordinate system. The position of the joint was manually optimized to best possible match bone's motions while avoiding disarticulation or bone collision.

Segments were manually posed in Maya ® to match their X-ray shadow in both biplanar views. This process was repeated for several key frames of the X-ray video recordings with cubic spline interpolation to produce smooth movements that closely approximated the recorded motion. The resulting data, describing the segmental and joint angular changes over time of all bony elements, were exported in CSV format to Matlab®. In Matlab® we then normalized them and computed mean and standard deviation (SD^s.d.^). Results are presented by anatomical axis.

#### Anatomical marker positioning under fluoroscopic control

**Figure 10:**
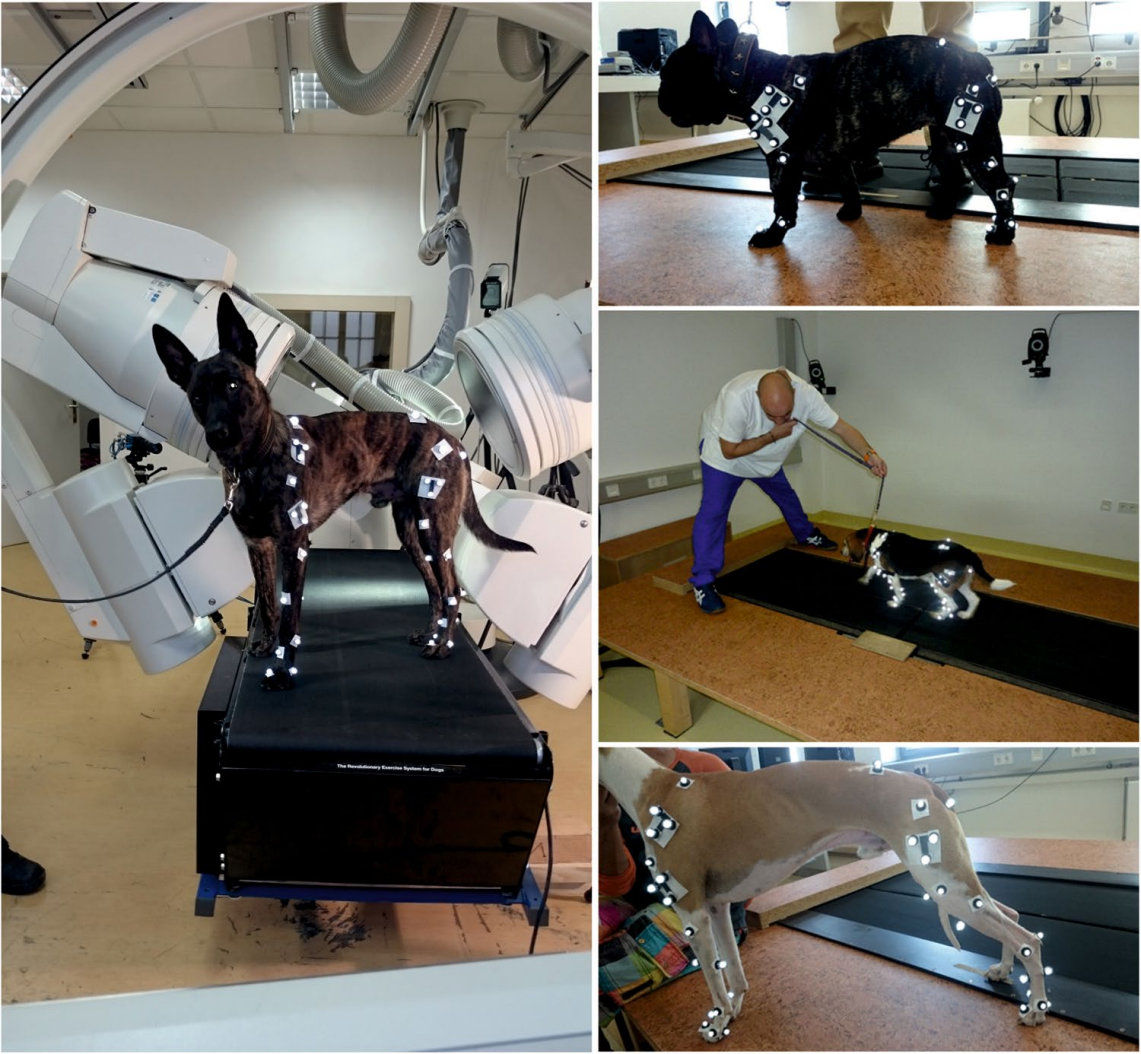
Jena X-ray Lab (left) and locomotion lab Hannover (right). All four breeds were measured in both labs. Marker were set under fluoroscopic control. Fluoroscopic data was used for Scientific Rotoscoping. Marker-based 3D kinematics were recorded at the locomotion lab in Hannover.

As described in^23^, the anatomical markers were positioned under fluoroscopic control (Fig. 10). Only the left pelvic limb of each dog was studied. Together with the anatomical markers, which were used to locate the proximal and distal centers of rotation, a cluster of three markers in the form of a T was attached to the femur (see Fig. 10). The hair was shaved from the site of each marker to ensure correct placement in the motion lab. In total, we attached 21 markers as follows: The markers were placed at the distal aspect of the second and fifth and the dorsal aspect of the third metatarsal bones, plantar aspect of the metatarsophalangeal joint, medial and lateral malleoli, caudal aspect of tibiotarsal (hock) joint, the medial and lateral condyles of the femur, greater trochanter of the left femur, left and right ischial tuberosities of the pelvis, the most dorsal aspect of the left and right ilial bodies of the pelvis, the dorsal and plantar aspects of the foot approximately midway between the metatarsophalangeal and tibiotarsal joints, cranial and caudolateral aspects of the tibia approximately midway between the hock and stifle joints, patella, and T-cluster of three marker at lateral aspect of the thigh approximately midway between the stifle and hip joints.

3D kinematic data were collected using 6 infrared Vicon® cameras (Oxford Metrics, Oxford, UK) and an instrumented quad-band treadmill (model 4060-08, Bertec Corporation) available at the locomotion lab of the Small Animal Hospital of the University of Veterinary Medicine Hannover, Germany. Kinematic data were collected at 100 Hz. Marker and T-cluster sites were shaved. Data collection started as soon as the dogs were walking or trotting comfortably. Data were recorded for a maximum of 45 s. For computation, series of at least 5 cycles (strides) were used in which the dog moved steadily and without overstepping onto the other bands (force plates) of the four-split treadmill. When trotting, dogs were kept on one side of the treadmill (usually the left side) to facilitate handling. The lab coordinate system was set as follows: +x pointed left, +y pointed opposite to the direction of motion and +z pointed upwards.

The 3D coordinates of marker trajectories were smoothed by a Butterworth four order low-pass filter with a cut-off frequency of 6 Hz. To obtain 3D angular kinematics we used the cardan sequence of three rotations about x, y, z axes^23,55^ (i.e. pro-/retraction, ad-/abduction, axial rotation).

1 $$[\begin{array}{c}{x}_{3}\\ {y}_{3}\\ {z}_{3}\end{array}]=[\begin{array}{ccc}{c}_{2}{c}_{3} & {s}_{3}{c}_{1}+{s}_{1}{s}_{2}{c}_{3} & {s}_{1}{s}_{3}-{c}_{1}{s}_{2}{c}_{3}\\ -{c}_{2}{c}_{3} & {c}_{1}{c}_{3}-{s}_{1}{s}_{2}{s}_{3} & {s}_{1}{c}_{3}+{c}_{1}{s}_{2}{s}_{3}\\ {s}_{2} & -{s}_{1}{c}_{2} & {c}_{1}{c}_{2}\end{array}]\,[\begin{array}{c}{x}_{0}\\ {y}_{0}\\ {z}_{0}\end{array}]$$

Note that (1) is transposed to obtain angles measured from lab coordinate system. The angles θ_1_, θ_2,_ θ_3_ were obtained from the trigonometric relationships present in the transformation matrix and are expressed as global coordinates. Joint angles were obtained by computing the rotation matrix between two adjacent segments, in turn obtained by transforming the coordinate system of the proximal segment into the coordinate system of the distal segment. For example, hip joint angles were obtained from the following transformation: [R_hip_] = [Fe][Pe]^T^, where R_hip_ is the transformation matrix and Fe and Pe the coordinate system belonging to femur and pelvis.

Hip joint position was estimated relative to the position of the four markers attached to the pelvis, and the hip joint center obtained from the virtual joint in SR.

The angles computed from marker data were transformed into the SR coordinate system by multiplying retraction/protraction and flexion/extension angles by −1. Afterwards, flexion-extension angles computed from marker data were transformed to match the reference pose by using the relation: fl-ext_rotoscoping_ = fl-ext_marker_ − 180° [°].

To compare the respective influence of gait and breed on joint angles, a repeated measures ANOVA with gait (walk vs. trot) as within subjects and breed (Whippet, French bulldog, Malinois and Beagle) as between subjects was performed. Post-hoc tests with Bonferroni correction were used to test for differences between breeds. We analyzed scientific rotoscoping data from pelvis, as the first element of the kinematic chain, the hip, and the stifle. Comparisons were done at specific timepoints (TD, midstance, TO, and midswing). Statistical analysis was performed in IBM SPSS 25 Chicago, IL, USA. Femoral and tibial segmental angles were used merely as an aid to better understand joint kinematics and, were not analyzed statistically.

## Electronic supplementary material

Supplementary information

## Data Availability

The datasets generated during and/or analyzed during the current study are available from the corresponding author on reasonable request. X-ray films were saved into the Jena collection of X-ray movies of the University of Jena, and can be accessed upon request.
