# Supplementary material for: Three-dimensional kinematics of canine hind limbs: in vivo, biplanar, high-frequency fluoroscopic analysis of four breeds during walking and trotting
Source: Sci Rep. 2018 Nov 19;8:16982. doi: 10.1038/s41598-018-34310-0 (PMC6242825; doi:10.1038/s41598-018-34310-0)
Supplement: Supplementary file 1 — Supplementary information [file 41598_2018_34310_MOESM1_ESM.pdf]

Three-dimensional kinematics of canine hind limbs: in vivo, biplanar, high-frequency fluoroscopic analysis of four breeds during walking and trotting

Martin S. Fischer, Silvia V. Lehmann, Emanuel Andrada

Institut für Zoologie und Evolutionsforschung, Friederich-Schiller-Universität Jena, Erbertstr. 1, 07743, Jena, Germany.

Corresponding author: Martin S. Fischer (martin.fischer@uni-jena.de)

Silvia V. Lehmann (silvia-veronica.lehmann@uni-jena.de)

Emanuel Andrada (emanuel.andrada@uni-jena.de)

Supplementary information: Mean values and standard deviation of segmental and joint kinematics at specific timepoints based on marker data.

Table S1. Segments and joints kinematics at walk during stance and swing phases based on marker data. Bread: Whippet.

	% Stance					% Swing		
	TD	25	50	75	TO	25	50	75
Pelvis_lat-med_ax_mean	45.6	44.3	45.6	49.7	47.6	45.2	43.2	43.9
Pelvis_lat-med_ax_std	2.9	3.9	3.9	2.7	3.5	4.0	3.8	3.1
Femur_lat-med_ax_mean	34.9	25.1	12.3	-0.1	-3.0	8.6	25.4	37.4
Femur_lat-med_ax_std	6.2	7.4	8.9	11.6	12.6	9.8	6.6	4.5
Tibia_lat-med_ax_mean	-12.8	-29.4	-42.8	-53.5	-60.3	-62.7	-44.3	-16.8
Tibia_lat-med_ax_std	6.2	4.9	4.9	5.1	5.5	5.4	10.0	9.0
Metatarsus_lat-med_ax_mean	43.9	35.7	19.7	2.9	-18.3	-3.2	25.0	40.0
Metatarsus_lat-med_ax_std	6.8	7.4	7.0	8.0	6.9	8.1	7.2	7.8
Pelvis_di-pro_ax_mean	-6.9	-3.2	2.6	4.9	3.4	1.5	-2.5	-5.9
Pelvis_di-pro_ax_std	2.1	4.0	3.4	4.1	3.2	3.1	3.5	2.7
Femur_di-pro_ax_mean	-6.4	-5.1	-6.1	-8.3	-7.9	-8.3	-7.4	-5.1
Femur_di-pro_ax_std	14.0	12.2	9.0	11.2	16.3	16.8	15.9	17.3
Tibia_di-pro_ax_mean	4.0	11.9	9.4	11.1	11.8	11.8	13.3	4.8
Tibia_di-pro_ax_std	17.9	9.7	6.2	6.0	6.4	6.4	8.9	19.4
Metatarsus_di-pro_ax_mean	3.6	3.9	5.5	9.7	14.5	10.6	7.2	7.9
Metatarsus_di-pro_ax_std	25.0	23.6	24.2	24.0	26.2	24.2	26.1	26.7
Pelvis_cr-cau_ax_mean	3.8	3.0	4.1	3.8	4.2	4.7	3.3	2.3
Pelvis_cr-cau_ax_std	3.6	3.0	3.7	3.5	3.8	3.8	3.6	3.9
Femur_cr-cau_ax_mean	-13.5	-13.9	-15.1	-15.7	-14.7	-18.6	-19.0	-17.8
Femur_cr-cau_ax_std	2.6	2.5	3.0	3.5	5.4	4.1	2.2	2.3
Tibia_cr-cau_ax_mean	-0.6	0.2	3.2	4.7	3.8	4.7	1.5	-0.7
Tibia_cr-cau_ax_std	6.0	5.3	4.8	4.5	4.4	4.0	4.2	5.7
Metatarsus_cr-cau_ax_mean	-7.4	-5.4	-4.5	-4.3	-4.9	-6.2	-7.5	-7.7
Metatarsus_cr-cau_ax_std	8.3	8.7	9.0	9.9	9.5	9.2	9.6	9.2
Hip_lat-med_ax_mean	-8.2	-17.1	-28.5	-39.3	-42.7	-33.5	-18.1	-7.2
Hip_lat-med_ax_std	1.9	2.8	4.4	4.9	6.4	6.0	4.8	2.3
Stifle_lat-med_ax_mean	-38.4	-45.2	-47.7	-47.3	-48.7	-61.8	-60.5	-42.7
Stifle_lat-med_ax_std	2.5	2.2	2.1	2.9	3.3	3.3	4.1	2.9
Hock_lat-med_ax_mean	49.1	55.6	53.2	47.8	33.7	50.2	60.3	46.4
Hock_lat-med_ax_std	3.9	1.8	2.2	2.7	3.1	5.4	4.3	4.8
Hip_di-pro_ax_mean	-16.5	-15.7	-12.8	-14.1	-17.9	-19.1	-17.0	-16.9
Hip_di-pro_ax_std	3.0	3.7	4.4	5.1	4.6	5.4	6.9	4.4
Stifle_di-pro_ax_mean	7.0	9.4	2.9	2.3	4.4	1.2	2.8	2.8
Stifle_di-pro_ax_std	20.7	13.7	11.2	11.6	12.5	10.3	11.1	21.8
Hock_di-pro_ax_mean	0.1	-1.2	-1.8	0.4	3.5	1.3	0.9	2.7
Hock_di-pro_ax_std	7.1	5.7	5.2	3.8	4.4	3.9	4.1	4.1
Hip_cr-cau_ax_mean	-4.0	-4.8	-5.3	-7.8	-7.6	-6.9	-7.7	-8.2
Hip_cr-cau_ax_std	5.4	5.0	6.6	7.5	7.3	6.8	5.7	5.1
Stifle_cr-cau_ax_mean	19.2	18.9	17.6	19.7	20.4	22.0	18.8	21.1
Stifle_cr-cau_ax_std	2.3	4.4	2.8	1.3	2.6	4.5	5.2	4.9
Hock_cr-cau_ax_mean	-0.6	2.6	1.9	2.3	0.5	2.8	5.0	2.8
Hock_cr-cau_ax_std	8.4	6.4	6.9	6.7	6.1	6.7	6.5	6.8

std: standard deviation, TD: touch-down, TO: toe-off, lat-med_ax: latero-medial axis, cr-au_ax: cranio-caudal axis, di-pro-ax: distal-proximal axis.

Table S2. Segments and joints kinematics at trot during stance and swing phases based on marker data. Bread: Whippets.

	% Stance					% Swing		
	TD	25	50	75	TO	25	50	75
Pelvis_lat-med_ax_mean	-40.2	-38.6	-38.6	-39.8	-40.6	-38.9	-40.0	-42.7
Pelvis_lat-med_ax_std	2.0	3.0	4.3	4.8	4.2	3.2	3.2	2.5
Femur_lat-med_ax_mean	-31.0	-24.4	-17.1	-7.8	1.3	-0.3	-23.8	-38.9
Femur_lat-med_ax_std	7.0	7.5	8.3	9.7	10.5	7.5	5.9	7.1
Tibia_lat-med_ax_mean	-18.5	-34.4	-48.1	-55.7	-57.9	-68.4	-70.1	-27.5
Tibia_lat-med_ax_std	3.9	4.0	4.1	4.3	4.5	5.6	8.5	8.8
Metatarsus_lat-med_ax_mean	38.7	36.8	26.0	10.6	-17.1	-8.7	12.7	38.3
Metatarsus_lat-med_ax_std	4.7	5.9	7.3	7.8	7.5	5.7	5.6	6.7
Pelvis_di-pro_ax_mean	5.8	5.0	2.9	1.0	-0.9	-1.6	2.4	5.2
Pelvis_di-pro_ax_std	4.8	3.1	1.7	2.0	3.4	3.5	2.3	4.7
Femur_di-pro_ax_mean	-11.5	-9.9	-7.8	-9.5	-13.4	-12.8	-8.0	-6.3
Femur_di-pro_ax_std	4.7	3.9	3.9	4.7	3.8	3.6	5.7	6.0
Tibia_di-pro_ax_mean	3.9	1.3	2.3	2.5	3.3	5.0	3.3	5.6
Tibia_di-pro_ax_std	3.1	2.4	2.8	2.8	2.6	5.2	3.5	4.3
Metatarsus_di-pro_ax_mean	-0.3	-4.9	-3.0	0.0	3.7	8.2	1.0	3.7
Metatarsus_di-pro_ax_std	7.3	7.3	6.2	6.6	7.1	7.4	6.8	7.7
Pelvis_cr-cau_ax_mean	-6.5	-7.1	-6.6	-6.5	-8.5	-9.5	-7.4	-7.4
Pelvis_cr-cau_ax_std	3.9	5.0	4.4	4.6	6.4	7.1	7.7	5.6
Femur_cr-cau_ax_mean	-7.8	-7.9	-9.7	-11.0	-9.1	-12.8	-10.1	-6.9
Femur_cr-cau_ax_std	3.8	3.8	3.5	2.1	2.4	4.5	5.8	4.6
Tibia_cr-cau_ax_mean	2.1	2.3	3.1	3.9	3.8	6.1	3.2	5.0
Tibia_cr-cau_ax_std	2.9	3.1	3.1	3.1	3.6	3.9	3.6	3.7
Metatarsus_cr-cau_ax_mean	0.8	2.6	3.4	3.5	2.6	1.4	2.0	2.2
Metatarsus_cr-cau_ax_std	3.4	2.9	2.9	2.8	2.9	3.5	3.8	4.0
Hip_lat-med_ax_mean	-8.1	-14.4	-20.9	-29.4	-38.1	-37.8	-14.6	-0.1
Hip_lat-med_ax_std	6.9	7.0	6.9	7.3	7.6	6.3	4.0	6.0
Stifle_lat-med_ax_mean	-46.8	-57.2	-65.2	-64.4	-57.5	-68.3	-82.5	-64.7
Stifle_lat-med_ax_std	10.6	11.4	11.6	13.1	14.3	13.8	5.0	14.8
Hock_lat-med_ax_mean	57.4	71.1	74.0	66.1	49.6	50.6	80.8	66.0
Hock_lat-med_ax_std	6.1	7.1	9.5	10.3	9.5	8.8	7.5	9.6
Hip_di-pro_ax_mean	-13.2	-12.4	-9.9	-10.7	-13.3	-13.9	-11.1	-8.8
Hip_di-pro_ax_std	6.7	6.4	6.0	7.0	6.8	7.5	6.8	7.2
Stifle_di-pro_ax_mean	7.1	2.1	-2.2	-3.3	-0.2	0.5	-2.5	4.1
Stifle_di-pro_ax_std	4.0	3.7	3.8	4.4	4.4	5.1	5.1	4.3
Hock_di-pro_ax_mean	-4.1	-7.6	-6.4	-4.3	-0.9	0.2	-2.3	-3.2
Hock_di-pro_ax_std	8.5	8.5	6.9	7.0	8.5	8.7	7.4	9.2
Hip_cr-cau_ax_mean	-2.3	-1.2	-3.6	-5.5	-5.7	-5.0	0.5	-1.8
Hip_cr-cau_ax_std	5.5	5.6	4.8	4.8	5.2	4.9	6.9	7.1
Stifle_cr-cau_ax_mean	17.2	15.8	14.8	16.1	18.1	20.3	14.4	17.2
Stifle_cr-cau_ax_std	6.2	5.7	5.4	4.8	3.1	7.7	7.5	9.3
Hock_cr-cau_ax_mean	2.6	2.8	4.5	4.0	2.5	1.2	4.1	4.6
Hock_cr-cau_ax_std	2.7	2.7	3.3	3.8	4.2	2.7	2.6	3.1

std: standard deviation, TD: touch-down. TO: toe-off, lat-med_ax: latero-medial axis, cr-au_ax: cranio-caudal axis, di-pro-ax: distalproximal axis.

Table S3. Mean and standard deviations of segment and joint kinematics at walk based on marker data. Bread: French bulldog.

	% Stance					% Swing		
	TD	25	50	75	TO	25	50	75
Pelvis_lat-med_ax_mean	43.7	45.4	47.2	50.3	46.5	43.1	43.7	44.0
Pelvis_lat-med_ax_std	5.5	5.8	6.8	7.1	7.9	7.3	7.0	6.3
Femur_lat-med_ax_mean	44.1	28.1	16.7	23.7	35.5	45.4	55.3	57.5
Femur_lat-med_ax_std	7.5	5.9	3.9	5.6	11.8	9.7	4.9	5.8
Tibia_lat-med_ax_mean	-7.6	-21.5	-38.8	-51.4	-55.9	-38.8	-15.7	-4.0
Tibia_lat-med_ax_std	6.5	8.8	13.6	13.2	8.1	19.8	18.9	8.2
Metatarsus_lat-med_ax_mean	40.4	31.7	13.4	-15.2	0.6	24.6	35.4	37.8
Metatarsus_lat-med_ax_std	6.0	9.2	10.8	16.5	17.8	15.0	7.0	6.8
Pelvis_di-pro_ax_mean	-6.8	3.3	8.3	5.2	-4.0	-6.9	-9.0	-9.9
Pelvis_di-pro_ax_std	10.2	8.0	5.4	5.0	7.3	7.7	8.6	9.6
Femur_di-pro_ax_mean	14.0	23.5	24.6	14.0	1.4	1.6	3.2	7.1
Femur_di-pro_ax_std	9.7	7.4	8.5	12.8	10.0	11.5	12.8	13.2
Tibia_di-pro_ax_mean	1.6	3.6	6.2	5.4	5.8	3.9	0.9	0.4
Tibia_di-pro_ax_std	2.0	3.9	6.1	3.9	3.9	2.4	1.4	1.8
Metatarsus_di-pro_ax_mean	8.2	10.7	15.0	15.7	12.8	9.3	5.4	6.1
Metatarsus_di-pro_ax_std	11.9	11.4	10.2	13.4	15.9	14.5	11.7	11.5
Pelvis_cr-cau_ax_mean	-0.1	-0.2	3.5	7.8	10.4	9.5	5.8	2.1
Pelvis_cr-cau_ax_std	8.2	10.3	9.5	12.2	11.8	11.2	9.2	7.0
Femur_cr-cau_ax_mean	-26.0	-29.1	-29.6	-30.7	-32.7	-31.8	-28.0	-25.5
Femur_cr-cau_ax_std	4.1	4.1	6.7	11.8	7.3	8.7	7.1	4.4
Tibia_cr-cau_ax_mean	9.2	12.3	16.3	15.9	16.7	13.9	8.5	6.9
Tibia_cr-cau_ax_std	6.0	8.0	9.3	6.6	6.2	6.0	5.1	6.0
Metatarsus_cr-cau_ax_mean	13.2	13.3	16.3	19.8	25.4	23.4	17.2	12.6
Metatarsus_cr-cau_ax_std	8.9	7.9	5.7	4.7	6.2	7.3	9.2	8.4
Hip_lat-med_ax_mean	-2.6	-21.3	-33.8	-36.3	-16.4	-3.6	4.5	4.6
Hip_lat-med_ax_std	4.5	3.9	5.3	8.9	14.7	12.8	7.2	3.9
Stifle_lat-med_ax_mean	-49.8	-53.5	-53.8	-57.1	-77.6	-85.2	-74.6	-53.4
Stifle_lat-med_ax_std	7.3	7.7	7.1	6.7	6.4	4.3	8.9	6.6
Hock_lat-med_ax_mean	46.0	53.1	50.4	34.1	49.0	62.4	55.4	43.4
Hock_lat-med_ax_std	6.3	4.5	8.9	9.3	20.2	10.4	14.0	10.2
Hip_di-pro_ax_mean	15.1	23.6	30.6	22.5	10.2	6.4	6.2	8.3
Hip_di-pro_ax_std	5.9	8.5	13.1	17.7	14.3	11.8	9.4	7.9
Stifle_di-pro_ax_mean	-12.4	-27.2	-30.5	-24.0	-17.0	-15.4	-11.7	-7.1
Stifle_di-pro_ax_std	12.5	10.3	8.4	9.6	9.8	9.3	9.7	11.0
Hock_di-pro_ax_mean	4.9	6.4	4.0	9.5	15.1	10.9	7.8	5.3
Hock_di-pro_ax_std	8.7	8.0	4.9	3.9	5.0	10.4	10.7	8.9
Hip_cr-cau_ax_mean	-35.1	-35.1	-29.0	-24.9	-25.3	-28.9	-32.8	-35.1
Hip_cr-cau_ax_std	3.7	3.4	3.5	5.7	6.2	4.8	4.4	4.0
Stifle_cr-cau_ax_mean	19.7	16.3	13.3	15.0	13.6	15.0	18.4	20.8
Stifle_cr-cau_ax_std	6.6	7.6	7.1	8.8	9.4	9.1	7.4	8.6
Hock_cr-cau_ax_mean	5.5	1.5	-0.1	2.4	12.2	13.4	10.7	8.1
Hock_cr-cau_ax_std	6.2	7.3	8.1	6.1	6.0	6.4	6.2	7.2

std: standard deviation, TD: touch-down. TO: toe-off. Lat-med_ax: latero-medial axis, cr-au_ax: cranio-caudal axis, di-pro-ax: distal-proximal axis.

Table S4. Mean and standard deviations of segment and joint kinematics at trot based on marker data. Bread: French bulldog.

	% Stance					% Swing		
	TD	25	50	75	TO	25	50	75
Pelvis_lat-med_ax_mean	38.6	39.0	41.0	43.6	44.4	41.1	39.1	38.7
Pelvis_lat-med_ax_std	5.9	7.3	8.0	7.0	6.2	5.8	5.4	5.4
Femur_lat-med_ax_mean	38.3	27.6	13.9	9.5	12.5	-22.5	-41.3	-44.9
Femur_lat-med_ax_std	7.9	8.3	7.7	7.7	9.5	10.7	9.2	7.6
Tibia_lat-med_ax_mean	-9.7	-27.1	--37.4	-42.9	-52.1	-65.2	-36.6	1.9
Tibia_lat-med_ax_std	5.8	5.3	3.9	3.5	4.3	5.2	8.2	5.7
metatarsus_lat-med_ax_mean	33.7	33.0	19.2	-5.9	-20.9	3.9	39.3	39.5
metatarsus_lat-med_ax_std	9.6	11.6	12.6	12.4	7.0	7.8	9.3	7.5
Pelvis_di-pro_ax_mean	-7.5	-4.5	1.0	5.9	7.6	7.6	1.0	-6.7
Pelvis_di-pro_ax_std	2.9	3.4	3.3	2.7	3.0	2.9	3.6	3.2
Femur_di-pro_ax_mean	12.0	11.6	16.7	22.0	21.4	14.4	16.4	15.0
Femur_di-pro_ax_std	8.3	12.4	14.9	14.3	11.5	9.7	7.2	6.9
Tibia_di-pro_ax_mean	-0.8	-0.2	-0.1	-0.7	-0.4	1.9	1.8	-1.1
Tibia_di-pro_ax_std	1.0	0.8	1.2	1.0	0.9	2.8	3.2	1.3
Metatarsus_di-pro_ax_mean	-0.9	1.0	3.1	8.6	14.2	6.7	4.2	-3.1
Metatarsus_di-pro_ax_std	5.3	4.8	3.7	4.1	8.2	10.7	8.3	4.2
Pelvis_cr-cau_ax_mean	-1.3	-4.2	-5.1	-4.8	1.0	8.6	12.3	8.0
Pelvis_cr-cau_ax_std	4.8	3.6	2.7	3.0	4.2	5.8	5.9	5.4
Femur_cr-cau_ax_mean	-18.3	-21.5	-23.0	-25.3	-26.3	-24.2	-15.3	-10.9
Femur_cr-cau_ax_std	4.1	3.1	5.7	8.0	6.7	3.2	4.5	3.1
Tibia_cr-cau_ax_mean	10.8	14.0	16.2	14.8	15.0	18.5	17.2	11.5
Tibia_cr-cau_ax_std	4.0	3.9	4.6	4.8	4.9	6.0	6.3	4.7
Metatarsus_cr-cau_ax_mean	15.4	14.0	14.1	14.3	16.5	26.3	27.9	18.7
Metatarsus_cr-cau_ax_std	4.9	4.6	4.7	4.1	3.5	4.5	5.0	4.0
Hip_lat-med_ax_mean	-0.1	-10.5	-21.7	-30.2	-30.2	-15.9	2.7	7.6
Hip_lat-med_ax_std	3.7	3.5	3.6	4.9	6.2	6.5	4.8	3.7
Stifle_lat-med_ax_mean	-43.7	-54.5	-58.4	-56.3	-59.8	-78.1	-68.8	-36.8
Stifle_lat-med_ax_std	10.7	9.2	6.1	6.6	9.0	8.5	7.3	9.0
Hock_lat-med_ax_mean	41.9	58.0	54.4	35.7	30.1	62.5	68.7	36.0
Hock_lat-med_ax_std	8.0	11.3	11.3	10.6	8.5	10.0	8.8	7.5
Hip_di-pro_ax_mean	7.8	8.6	15.0	20.4	18.8	11.9	13.1	11.1
Hip_di-pro_ax_std	7.2	4.8	4.5	6.0	7.8	9.0	11.1	10.7
Stifle_di-pro_ax_mean	-5.9	-13.8	-22.0	-25.7	-21.0	-9.1	-5.3	-1.6
Stifle_di-pro_ax_std	6.7	7.1	7.4	7.4	6.4	5.1	5.5	7.3
Hock_di-pro_ax_mean	4.9	6.3	5.9	4.0	6.3	14.8	12.5	6.8
Hock_di-pro_ax_std	8.6	11.0	11.7	6.2	7.1	10.3	12.1	9.1
Hip_cr-cau_ax_mean	-21.8	-24.4	-26.0	-26.1	-21.4	-14.5	-11.4	-14.5
Hip_cr-cau_ax_std	3.2	2.9	3.0	4.1	4.4	2.8	3.8	3.3
Stifle_cr-cau_ax_mean	24.8	25.2	22.6	18.5	12.0	6.3	8.6	19.2
Stifle_cr-cau_ax_std	3.4	3.3	3.3	3.5	4.8	6.1	6.6	5.4
Hock_cr-cau_ax_mean	7.9	6.4	4.4	2.4	3.4	16.0	20.0	10.3
Hock_cr-cau_ax_std	6.4	6.5	6.8	6.1	7.0	8.2	9.4	6.1

std: standard deviation, TD: touch-down, TO: toe-off, lat-med_ax: latero-medial axis, cr-au_ax: cranio-caudal axis, di-pro-ax: ventro-dorsal axis.

Table S5. Mean and standard deviations of segment and joint kinematics at walk based on marker data. Bread: Malinois.

	% Stance					% Swing		
	TD	25	50	75	TO	25	50	75
Pelvis_lat-med_ax_mean	43.8	43.4	42.2	45.3	51.7	46.5	43.1	43.9
Pelvis_lat-med_ax_std	5.6	4.0	2.3	3.0	4.6	3.8	2.8	3.2
Femur_lat-med_ax_mean	41.1	30.1	15.7	3.0	7.0	2.1	24.5	41.5
Femur_lat-med_ax_std	5.2	5.2	5.1	6.6	8.1	4.8	3.8	4.7
Tibia_lat-med_ax_mean	-8.9	-26.9	-40.6	-52.1	-56.8	-62.5	-58.3	-22.0
Tibia_lat-med_ax_std	4.0	4.1	3.9	3.5	3.1	4.8	5.6	6.7
Metatarsus_lat-med_ax_mean	37.3	30.8	12.2	-7.0	-29.2	-28.9	4.6	31.7
Metatarsus_lat-med_ax_std	3.2	3.1	2.5	5.0	5.8	8.6	8.0	3.6
Pelvis_di-pro_ax_mean	-7.8	-2.4	2.5	5.4	3.1	0.4	-3.7	-6.4
Pelvis_di-pro_ax_std	2.6	2.8	2.6	3.3	3.8	4.0	4.5	3.3
Femur_di-pro_ax_mean	-2.1	2.7	3.2	-2.2	-17.2	-16.9	-12.2	-3.8
Femur_di-pro_ax_std	9.3	11.8	11.9	15.6	12.6	12.8	12.1	12.7
Tibia_di-pro_ax_mean	8.6	9.4	9.9	10.0	9.8	7.3	9.5	9.8
Tibia_di-pro_ax_std	13.5	12.8	12.3	11.8	12.7	12.6	13.0	11.4
Metatarsus_di-pro_ax_mean	8.1	8.7	11.6	14.8	17.4	18.2	16.0	13.2
Metatarsus_di-pro_ax_std	5.0	5.2	5.9	7.2	9.4	10.2	6.7	4.5
Pelvis_cr-cau_ax_mean	3.9	3.7	1.9	4.7	5.7	8.8	8.6	6.3
Pelvis_cr-cau_ax_std	3.7	3.1	3.6	2.9	3.6	5.5	3.2	2.6
Femur_cr-cau_ax_mean	-13.5	-11.7	-14.4	-15.8	-12.5	-16.1	-15.7	-14.1
Femur_cr-cau_ax_std	5.9	5.9	5.3	6.7	8.6	9.7	8.2	5.8
Tibia_cr-cau_ax_mean	3.3	6.8	8.9	9.3	7.1	7.8	9.4	7.2
Tibia_cr-cau_ax_std	3.0	2.3	1.9	1.7	5.3	6.6	5.1	3.0
Metatarsus_cr-cau_ax_mean	-6.5	-7.0	-5.8	-4.8	-2.7	-1.5	-4.5	-5.5
Metatarsus_cr-cau_ax_std	5.5	6.6	6.6	6.9	7.0	6.5	7.1	6.3
Hip_lat-med_ax_mean	1.6	-7.2	-21.8	-33.1	-43.0	-34.8	-12.9	3.1
Hip_lat-med_ax_std	6.9	7.8	9.1	8.1	7.7	5.5	5.7	6.7
Stifle_lat-med_ax_mean	-49.1	-60.1	-61.2	-61.1	-54.6	-67.7	-84.7	-65.6
Stifle_lat-med_ax_std	4.7	7.8	6.8	8.4	9.5	8.7	4.0	10.3
Hock_lat-med_ax_mean	46.4	58.9	54.3	47.0	30.2	35.7	64.6	54.5
Hock_lat-med_ax_std	5.1	6.6	4.3	4.2	4.0	7.4	5.0	6.9
Hip_di-pro_ax_mean	1.1	6.4	11.8	10.4	0.2	-3.5	-4.8	-0.3
Hip_di-pro_ax_std	9.9	10.9	12.2	15.3	14.9	15.2	13.6	12.1
Stifle_di-pro_ax_mean	0.8	-2.1	-6.1	-4.4	1.4	-1.1	-1.0	0.4
Stifle_di-pro_ax_std	9.9	12.8	11.2	9.6	9.7	11.7	14.7	10.7
Hock_di-pro_ax_mean	-0.3	-1.4	-0.8	1.8	7.5	9.5	4.7	2.9
Hock_di-pro_ax_std	4.8	2.8	3.1	4.7	7.6	7.0	1.8	3.6
Hip_cr-cau_ax_mean	-9.4	-8.3	-9.4	-8.1	-8.3	-6.5	-6.8	-10.2
Hip_cr-cau_ax_std	3.4	3.3	4.5	3.6	4.1	3.8	2.0	3.2
Stifle_cr-cau_ax_mean	7.2	4.8	4.0	5.2	11.8	11.6	11.4	10.1
Stifle_cr-cau_ax_std	7.0	4.6	3.4	2.4	4.2	3.0	4.7	6.6
Hock_cr-cau_ax_mean	0.3	0.4	0.1	-0.4	0.1	0.5	2.7	1.9
Hock_cr-cau_ax_std	7.2	6.1	6.4	5.7	4.1	4.3	5.7	6.7

std: standard deviation, TD: touch-down, TO: toe-off, lat-med_ax: latero-medial axis, cr-au_ax: cranio-caudal axis, di-pro-ax: distal-proximal axis.

Table S6. Mean and standard deviations of segment and joint kinematics at trot based on marker data. Bread: Malinois.

	% Stance					% Swing		
	TD	25	50	75	TO	25	50	75
Pelvis_lat-med_ax_mean	36.7	37.1	40.3	43.7	45.3	40.1	37.6	37.2
Pelvis_lat-med_ax_std	3.3	3.6	4.6	4.5	3.7	3.1	3.9	4.1
Femur_lat-med_ax_mean	31.0	16.5	3.4	-7.1	-6.4	6.0	24.4	36.9
Femur_lat-med_ax_std	4.0	4.4	3.6	3.6	3.7	4.1	3.7	3.8
Tibia_lat-med_ax_mean	-15.2	--37.8	-51.5	-53.6	-60.7	-72.1	-62.1	-21.0
Tibia_lat-med_ax_std	2.6	1.9	2.9	2.7	2.7	3.9	6.0	4.9
metatarsus_lat-med_ax_mean	31.6	26.5	4.0	-22.4	-33.6	-18.4	13.6	32.4
metatarsus_lat-med_ax_std	4.5	5.2	4.6	3.9	2.6	3.3	3.6	5.8
Pelvis_di-pro_ax_mean	-4.1	-0.7	3.9	6.2	6.9	4.6	-0.4	-2.6
Pelvis_di-pro_ax_std	2.8	3.5	2.9	2.6	2.6	3.4	5.1	3.5
Femur_di-pro_ax_mean	0.9	-5.1	-7.1	-14.2	-14.7	-16.9	-4.8	3.8
Femur_di-pro_ax_std	9.9	16.9	17.8	14.1	12.2	12.4	13.0	8.4
Tibia_di-pro_ax_mean	5.8	2.8	2.1	3.3	2.4	1.4	-0.9	9.5
Tibia_di-pro_ax_std	12.4	11.4	11.6	10.5	8.1	12.7	12.4	14.1
Metatarsus_di-pro_ax_mean	6.4	6.5	6.8	10.2	11.6	10.2	10.3	14.4
Metatarsus_di-pro_ax_std	4.0	3.8	3.7	2.8	4.7	5.8	3.8	5.3
Pelvis_cr-cau_ax_mean	0.1	2.7	5.2	6.2	9.5	10.5	9.4	6.4
Pelvis_cr-cau_ax_std	3.7	3.5	3.8	2.8	3.3	2.4	3.2	3.1
Femur_cr-cau_ax_mean	-8.8	-6.7	-7.8	-7.3	-10.0	-11.3	-9.6	-8.8
Femur_cr-cau_ax_std	3.8	3.5	5.8	8.0	7.3	5.3	3.0	5.0
Tibia_cr-cau_ax_mean	6.6	9.2	8.5	5.4	4.9	4.9	8.0	11.8
Tibia_cr-cau_ax_std	3.1	4.7	3.6	2.1	2.2	4.2	4.1	4.0
Metatarsus_cr-cau_ax_mean	-0.1	0.3	1.9	3.7	4.6	4.8	6.1	1.8
Metatarsus_cr-cau_ax_std	1.6	3.4	3.2	1.7	1.1	2.6	5.0	2.5
Hip_lat-med_ax_mean	-4.3	-17.7	-29.8	-41.7	-41.5	-28.9	-7.8	2.9
Hip_lat-med_ax_std	6.4	7.4	7.5	6.5	6.8	6.4	4.6	5.3
Stifle_lat-med_ax_mean	-41.5	-49.6	-51.0	-41.5	-49.4	-72.5	-81.7	-54.5
Stifle_lat-med_ax_std	3.4	5.5	7.1	5.8	3.8	3.5	3.9	7.6
Hock_lat-med_ax_mean	46.0	63.7	53.4	30.3	27.0	52.5	73.5	51.9
Hock_lat-med_ax_std	4.7	5.6	9.5	5.8	4.9	6.1	7.3	9.1
Hip_di-pro_ax_mean	4.6	2.2	4.6	3.0	1.9	-6.0	-0.1	2.3
Hip_di-pro_ax_std	4.4	8.5	9.2	8.1	7.3	10.0	7.2	5.8
Stifle_di-pro_ax_mean	-1.4	1.7	0.6	3.0	2.4	2.5	-2.0	3.5
Stifle_di-pro_ax_std	6.5	4.1	5.0	5.9	3.7	11.2	12.5	10.2
Hock_di-pro_ax_mean	-1.9	-2.0	-0.5	5.1	7.6	5.4	3.6	1.5
Hock_di-pro_ax_std	5.5	4.6	4.6	8.1	8.2	4.9	2.4	5.6
Hip_cr-cau_ax_mean	-8.0	-3.8	-3.6	-4.0	-2.9	-1.3	-1.7	-6.5
Hip_cr-cau_ax_std	2.0	2.0	1.7	3.0	4.0	3.2	3.4	3.3
Stifle_cr-cau_ax_mean	4.2	5.6	2.9	3.9	3.8	8.2	4.3	7.2
Stifle_cr-cau_ax_std	7.1	5.3	4.8	4.1	4.0	7.3	3.8	10.1
Hock_cr-cau_ax_mean	2.1	1.6	1.5	3.8	4.3	5.5	5.5	5.3
Hock_cr-cau_ax_std	6.3	6.8	5.0	2.7	2.4	6.2	5.4	8.1

std: standard deviation, TD: touch-down, TO: toe-off, lat-med_ax: latero-medial axis. cr-au_ax: cranio-caudal axis, di-pro-ax: ventro-dorsal axis.

Table S7. Mean and standard deviations of segment and joint kinematics at walk based on marker data. Bread: Beagle.

	% Stance					% Swing		
	TD	25	50	75	TO	25	50	75
Pelvis_lat-med_ax_mean	35.7	34.9	32.1	38.7	39.4	33.3	29.7	31.5
Pelvis_lat-med_ax_std	5.2	6.4	4.6	3.8	6.0	5.2	4.6	4.7
Femur_lat-med_ax_mean	41.3	32.7	21.7	14.9	10.4	14.8	26.8	38.7
Femur_lat-med_ax_std	8.0	9.8	10.9	10.5	11.1	9.2	6.8	6.2
Tibia_lat-med_ax_mean	-3.3	-24.2	-36.1	-47.5	-52.7	-61.5	-50.4	-11.7
Tibia_lat-med_ax_std	3.7	4.1	4.8	6.0	2.5	4.2	11.5	8.1
Metatarsus_lat-med_ax_mean	44.2	35.2	11.5	-8.9	-30.3	-22.7	11.4	34.2
Metatarsus_lat-med_ax_std	10.3	8.8	11.6	13.1	6.9	8.5	5.9	8.6
Pelvis_di-pro_ax_mean	-5.7	0.7	6.1	5.6	1.7	-3.2	-6.1	-7.3
Pelvis_di-pro_ax_std	3.4	4.8	3.9	4.7	5.9	3.9	4.5	4.8
Femur_di-pro_ax_mean	0.3	0.3	1.1	-1.8	-4.9	-6.1	-5.1	-3.4
Femur_di-pro_ax_std	8.4	6.1	7.0	9.0	10.3	10.3	8.7	9.5
Tibia_di-pro_ax_mean	-5.1	-3.1	-2.5	-3.0	-3.3	-5.8	-7.8	-8.1
Tibia_di-pro_ax_std	9.8	8.5	10.8	10.5	10.4	9.5	9.0	10.8
Metatarsus_di-pro_ax_mean	-7.2	-6.5	-1.4	-1.8	2.4	2.6	-3.3	-5.6
Metatarsus_di-pro_ax_std	8.4	7.4	8.5	7.6	9.3	8.2	7.2	9.3
Pelvis_cr-cau_ax_mean	13.7	11.9	9.7	9.6	9.8	13.1	13.6	13.2
Pelvis_cr-cau_ax_std	4.4	5.5	5.0	4.7	5.4	4.9	5.4	6.1
Femur_cr-cau_ax_mean	-13.7	-16.4	-17.7	-20.1	-21.2	-18.2	-16.1	-15.3
Femur_cr-cau_ax_std	8.5	9.7	8.7	8.0	8.2	10.1	10.0	9.1
Tibia_cr-cau_ax_mean	-1.8	2.1	2.9	3.0	2.4	0.1	-4.7	-4.6
Tibia_cr-cau_ax_std	6.9	6.2	6.0	7.3	8.6	5.3	2.8	5.5
Metatarsus_cr-cau_ax_mean	-0.8	-0.7	2.5	3.6	3.8	0.9	3.3	0.2
Metatarsus_cr-cau_ax_std	6.0	3.5	3.3	2.1	2.3	3.6	3.5	3.9
Hip_lat-med_ax_mean	7.1	-2.3	-14.4	-22.5	-27.8	-22.0	-8.6	4.5
Hip_lat-med_ax_std	6.0	9.0	11.6	12.0	14.8	14.6	9.5	5.1
Stifle_lat-med_ax_mean	-48.4	-60.5	-62.0	-66.2	-67.6	-78.3	-76.0	-51.8
Stifle_lat-med_ax_std	9.2	11.3	12.1	12.5	10.4	8.4	11.7	12.6
Hock_lat-med_ax_mean	49.0	57.5	46.9	35.8	26.2	52.1	65.3	47.1
Hock_lat-med_ax_std	14.9	7.4	7.6	7.8	10.1	9.7	8.2	11.5
Hip_di-pro_ax_mean	-1.8	0.6	1.7	-1.7	-5.4	-7.1	-7.5	-7.2
Hip_di-pro_ax_std	5.6	6.4	7.0	7.7	7.2	7.6	7.1	6.3
Stifle_di-pro_ax_mean	-14.2	-17.0	-18.6	-20.0	-20.8	-22.3	-19.8	-14.8
Stifle_di-pro_ax_std	14.3	12.3	11.6	11.1	9.1	9.3	13.5	17.5
Hock_di-pro_ax_mean	-6.9	-4.1	3.0	6.7	13.8	12.4	1.7	-5.5
Hock_di-pro_ax_std	11.3	11.8	11.4	13.6	15.4	13.1	12.4	10.6
Hip_cr-cau_ax_mean	-11.8	-14.8	-16.4	-18.3	-19.0	-16.3	-14.6	-13.3
Hip_cr-cau_ax_std	8.2	6.0	4.9	5.3	4.8	5.3	6.5	7.7
Stifle_cr-cau_ax_mean	0.5	3.4	6.0	9.9	11.0	9.1	5.5	2.7
Stifle_cr-cau_ax_std	6.5	6.6	7.1	8.1	7.0	6.6	5.8	7.1
Hock_cr-cau_ax_mean	-3.4	-6.3	-4.0	-2.1	-0.2	-5.6	-2.8	-1.0
Hock_cr-cau_ax_std	11.0	8.0	7.4	6.6	6.2	5.7	7.1	10.9

std: standard deviation, TD: touch-down, TO: toe-off, lat-med_ax: latero-medial axis, cr-au_ax: cranio-caudal axis, di-pro-ax: distal-proximal axis.

Table S8. Mean and standard deviations of segment and joint kinematics at trot based on marker data. Bread: Beagle.

	% Stance					% Swing		
	TD	25	50	75	TO	25	50	75
Pelvis_lat-med_ax_mean	25.9	24.1	24.1	27.0	30.6	30.1	23.0	26.7
Pelvis_lat-med_ax_std	1.8	2.2	3.0	2.7	2.2	2.2	2.8	2.2
Femur_lat-med_ax_mean	30.9	22.1	13.4	9.2	6.7	9.5	24.2	34.1
Femur_lat-med_ax_std	6.6	7.9	8.9	9.6	10.4	11.2	8.8	6.4
Tibia_lat-med_ax_mean	-11.4	-29.7	-43.0	-48.4	--51.2	-69.1	-67.2	-20.4
Tibia_lat-med_ax_std	6.3	5.4	5.0	5.4	4.2	5.6	6.7	8.1
Metatarsus_lat-med_ax_mean	30.0	27.5	13.8	-9.5	-29.2	-32.7	2.2	26.4
metatarsus_lat-med_ax_std	10.7	9.4	9.4	9.7	8.1	9.6	9.6	11.6
Pelvis_di-pro_ax_mean	-8.8	-6.0	-1.3	2.8	4.8	3.6	-1.2	-7.8
Pelvis_di-pro_ax_std	4.3	4.2	3.9	4.6	4.8	3.6	4.6	4.7
Femur_di-pro_ax_mean	-2.4	-4.2	-5.9	-6.4	-6.6	-9.7	-6.6	-4.7
Femur_di-pro_ax_std	1.9	1.4	1.3	1.5	1.6	3.9	1.6	2.2
Tibia_di-pro_ax_mean	-3.0	-4.2	-4.7	-3.5	-1.7	-4.1	-7.7	-6.1
Tibia_di-pro_ax_std	10.1	9.0	9.1	9.0	8.4	7.4	7.9	9.2
Metatarsus_di-pro_ax_mean	-0.7	-1.6	-2.2	0.3	5.5	7.2	1.5	1.1
Metatarsus_di-pro_ax_std	9.6	9.1	9.4	12.5	14.1	17.1	12.5	11.0
Pelvis_cr-cau_ax_mean	2.3	3.4	5.5	5.4	6.2	10.2	8.5	8.1
Pelvis_cr-cau_ax_std	3.6	3.2	3.0	2.0	2.0	3.7	3.8	4.6
Femur_cr-cau_ax_mean	-4.3	-4.8	-6.1	-8.9	-12.3	-11.0	-6.0	-4.7
Femur_cr-cau_ax_std	6.0	4.1	4.3	4.8	4.9	3.1	2.9	5.2
Tibia_cr-cau_ax_mean	-1.9	-0.5	0.2	-0.2	-0.1	2.6	-1.6	-0.3
Tibia_cr-cau_ax_std	5.0	4.2	3.9	3.3	4.1	2.7	6.1	4.5
Metatarsus_cr-cau_ax_mean	-1.1	-1.8	-2.2	-1.5	0.5	2.6	4.9	3.1
Metatarsus_cr-cau_ax_std	2.5	1.7	2.3	2.6	2.5	2.9	4.7	3.9
Hip_lat-med_ax_mean	3.4	-5.2	-13.3	-16.9	-18.8	-14.8	-1.4	6.1
Hip_lat-med_ax_std	5.7	6.6	7.1	7.1	7.2	5.5	5.2	5.3
Stifle_lat-med_ax_mean	-42.0	-51.6	-56.4	-57.5	-57.8	-73.5	-81.8	-54.1
Stifle_lat-med_ax_std	10.3	9.7	9.3	8.1	9.1	10.6	5.3	10.2
Hock_lat-med_ax_mean	52.4	57.4	49.2	41.0	27.5	52.9	82.8	53.4
Hock_lat-med_ax_std	10.3	10.3	14.2	10.5	7.4	4.4	4.8	7.7
Hip_di-pro_ax_mean	-0.7	-1.2	-2.3	-2.7	-3.0	-7.9	-3.1	-3.3
Hip_di-pro_ax_std	8.6	8.1	9.2	9.1	9.2	10.0	7.9	9.3
Stifle_di-pro_ax_mean	-4.6	-5.5	-6.7	-7.8	-8.9	-13.6	-13.5	-7.4
Stifle_di-pro_ax_std	9.5	8.2	8.0	7.7	7.1	7.4	7.7	7.5
Hock_di-pro_ax_mean	-2.6	-4.7	-5.4	-3.4	2.2	2.5	-2.3	-2.9
Hock_di-pro_ax_std	8.3	8.1	7.7	8.9	8.0	7.7	8.7	9.5
Hip_cr-cau_ax_mean	-13.3	-12.0	-12.9	-15.8	-18.9	-16.2	-11.7	-13.4
Hip_cr-cau_ax_std	2.7	2.4	2.6	3.4	4.3	2.7	2.3	1.4
Stifle_cr-cau_ax_mean	2.6	5.5	8.1	9.7	11.6	13.8	4.8	5.9
Stifle_cr-cau_ax_std	6.3	5.6	5.3	5.1	4.9	3.3	4.7	6.9
Hock_cr-cau_ax_mean	-1.8	-4.6	-5.6	-3.8	-0.2	-1.8	0.0	-0.4
Hock_cr-cau_ax_std	6.7	5.2	4.1	4.9	5.2	5.7	5.7	7.3

std: standard deviation, TD: touch-down, TO: toe-off, lat-med_ax: latero-medial axis. cr-cau_ax: cranio-caudal axis. di-pro-ax: distal-proximal axis.
